# Age at menarche in South Asia: an interplay of sociodemographic, nutritional, lifestyle, anthropometric, biological, and environmental factors—a systematic review

**DOI:** 10.3389/fpubh.2026.1836422

**Published:** 2026-07-15

**Authors:** Anuja Amit Mohile, Rupali Tushar Waghode, Yaashodaa Kedar Padhye, Nalini R. Khatwani, Archana Ainapure, Radhika Prashant Hedaoo

**Affiliations:** 1 Symbiosis School of Culinary Arts and Nutritional Sciences, Symbiosis International (Deemed University), Pune, Maharashtra, India; 2 Texas School of Mental Health, Pune, Maharashtra, India; 3 Independent Researcher, Flower Mound, TX, United States; 4 Symbiosis International (Deemed University), Pune, Maharashtra, India; 5 Symbiosis Institute of International Business, Symbiosis International (Deemed University), Pune, Maharashtra, India; 6 Rising India Research Foundation, Pune, Maharashtra, India

**Keywords:** age at menarche, anthropometry, female adolescent, nutrition and lifestyle factors, nutritional indices, sociodemographic factors, South Asia

## Abstract

**Background:**

Age at menarche is a dynamic indicator of a female's health trajectories. Despite its relevance, the cohesive synthesis of its multifactorial determinants in South Asia is lacking, limiting the targeted public health strategies. This review examines the sociodemographic, nutritional, lifestyle, anthropometric, biological, and environmental determinants of age at menarche among females aged 8–21 years across South Asia.

**Methods:**

A systematic review of observational studies on determinants of age at menarche was conducted. Adhering to PRISMA guidelines, articles published between January 2014 and May 2025 were identified from ScienceDirect, PubMed, Web of Science, and Google Scholar. Methodological quality of eligible studies was assessed using the Joanna Briggs Institute Critical Appraisal Checklist. Due to significant heterogeneity across the studies, findings were narratively synthesized precluding meta-analysis.

**Results:**

Out of 729 articles, 18 studies from South Asian countries were included. The mean age at menarche clustered between 12 and 13 years across the studies. Menarche <12 years of age was associated with urban residence, a higher socioeconomic status, higher BMI, inadequate sleep and physical activity, higher consumption of fruits, vegetables, and non-vegetarian diet, excessive screen time, low birth weight, absence of biological father, mothers' age at menarche and exposure to environmental factors. On the contrary, the age of menarche >14 years of age was associated with rural residence, lower socioeconomic status, stunting and underweight.

**Conclusion:**

While heredity, demographics, and early-life programming influence age at menarche, modifiable nutrition and lifestyle factors are critical determinants in South Asia. Targeted interventions focusing on these determinants could reduce the burden of metabolic and growth-related disorders among adolescent females. Paucity of studies in South Asian countries is a critical evidence gap that needs attention to better inform policies for adolescent health.

**Systematic Review Registration:**

INPLASY, Identifier: INPLASY202610051.

## 1 Introduction

Menarche marks the reproductive maturity for female adolescents and is a sensitive indicator of fertility, overall health and nutritional status (1). Worldwide, the normal range of age at menarche is observed between 10 and 16 years, with the mean age ranging between 12 and 13 years (2). In South Asia, the mean age at menarche indicates notable variation, ranging from above 16 years in Nepal, over 13 years in Sri Lanka, around 15 years in Bangladesh and approximately 13.5 years in India. On the contrary, the mean age at menarche in various regions of the globe is <12 years. This finding indicates a substantial geographical variability in menarcheal timings. While there are established secular declines in developed countries, South Asia presents inconsistent evidence (3).

An early or late age at menarche is associated with significant long-term health complications (4). Evidence suggests that early age at menarche is associated with an increased exposure to estrogen and progesterone, predisposing women to breast and endometrial cancers, obesity, and cardiometabolic disorders such as type 2 diabetes (5, 6). Moreover, pre-term birth, shorter period of gestation, hypertension, and diabetes during pregnancy were also linked to the early menarcheal status of women (7, 8). Likewise, delayed menarche, which is connected with undernutrition, chronic illness, or high physical stress, is associated with osteoporosis, poor bone mineral density, subfertility, and psychosocial stress (9–12).

The age at menarche serves as a composite indicator of social transitions, health, and nutritional status among the population (13). It indicates the collective impact of physical activity, effects of sleep, nutrition, and psychosocial wellbeing (14). Declined age at menarche among over-nourished girls and undernourished girls, showing a typical delayed pattern of occurrence of menarche, emphasizes the triple burden of malnutrition across the globe, thereby highlighting the nutritional transition and challenge across the South Asian nations (9, 15).

In addition to social, nutritional, and lifestyle transition, biological factors, specifically heredity, are also identified as a strong influencing factor that determines the age at menarche. It is evidenced by girls following their mothers' or sisters' menarcheal age (16). Early life environment, specifically during the developmental phase, certain factors such as maternal age, nutritional status, prenatal nutrition, and birth order significantly impact birth weight and fetal development, eventually affecting the age at menarche (17, 18). Exposure to a poor environment, including household use of smoking, alcohol, and tobacco, as well as climate change, environmental toxins and endocrine-disrupting chemicals, has been identified as a key driver of menarcheal timings (13, 19). Although the data is evolving and pointing towards these emergent factors, the evidence is scattered in South Asia with varying differences in the age at menarche. Due to this scattered and diverse evidence on determinants of age at menarche, drawing a definite conclusion and making regional comparisons of various attributing factors is difficult. This hinders the development of targeted public health strategies. These observed differentials highlight a complex interplay of sociodemographic, nutritional, lifestyle, biological and environmental factors affecting the age of menarche (20).

Thus, within South Asian settings, there appears to be an uneven distribution of how nutrition and sociodemographic transitions are taking place. This further encumbers any clarity regarding the age at menarche, its determinants, and the health trajectories associated with it. Although the health implications associated with the discrepancies in the timings of menarche are evidenced, the age at menarche is not recognized and recommended as an indicator within the national adolescent health programs, nutrition monitoring and surveillance programs or any school health programs.

Worldwide, the global policy frameworks implicitly recognize the determinants of pubertal timing, such as “The WHO Life Course Approach to Nutrition” recognizes the impact of nutrition from early childhood through adolescence on growth, development and long-term health outcomes (21). Likewise, the UNICEF Adolescent Nutrition Framework affirms adolescence as a critical milestone for nutritional interventions for girls, considering its implications for reproductive health and intergenerational outcomes (22). In lower middle-income countries in South Asia, such as Bangladesh, Pakistan, Nepal and India, the focus is towards the anemia prevention, adolescent nutrition, sexual and reproductive health through their various national programs, such as Rashtriya Kishor Swasthya Karyakram, Adolescent Health Strategy (Bangladesh), National Nutrition Survey (Pakistan), and Adolescent Sexual and Reproductive Health Strategy (Nepal) (23–26). Rather than having separate vertical initiatives in parallel, age at menarche, alongside other focused outcomes, should also be considered an indicator in these adolescent-focused programs (27).

Although menstrual health and hygiene are addressed through programs such as Bangladesh's National Strategy on Menstrual Hygiene Management, Nepal's WASH in Schools standards and the emerging Dignified Menstruation Policy, and Pakistan's menstrual health and hygiene monitoring assessment and adolescent nutrition strategy and operational plan and India's Menstrual hygiene scheme, age at menarche is not integrated and monitored or addressed within these schemes, representing a critical gap in linking menstrual health programming with biological maturation and adolescent nutritional status (28–33).

**Figure 1:**
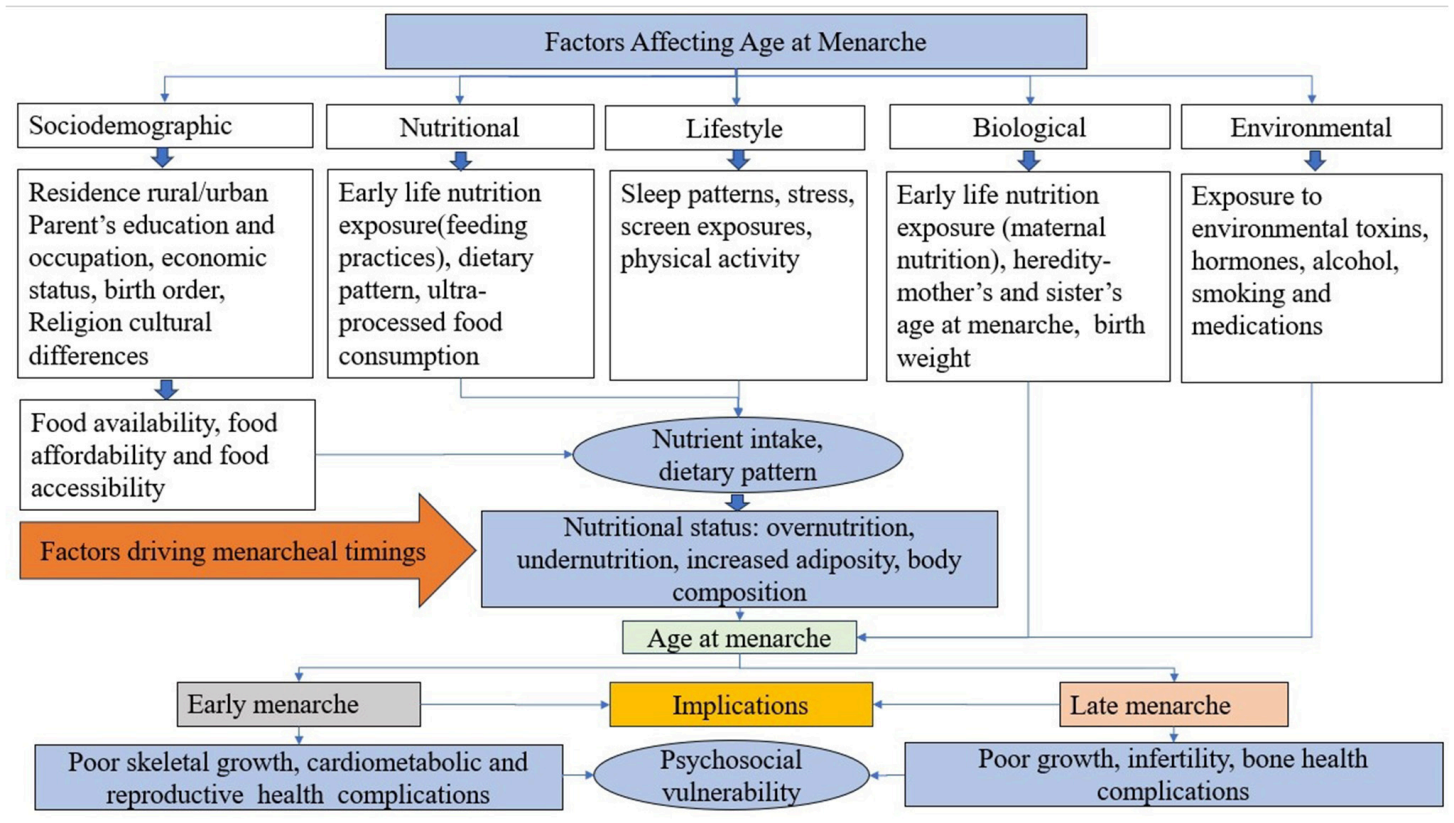
Conceptual framework illustrating the multidimensional determinants of age at menarche and their potential health consequences implications across the life course.

The conceptual framework (Figure 1) illustrates the multidimensional factors affecting the age at menarche, collected in this review, along with the possible future health implications, not assessed directly in this systematic review. The emerging gaps observed in the systematic review will provide organized evidence for developing targeted interventions and strategies to support adolescent health in future.

Hence, this comprehensive review undertakes a critical synthesis of existing research studies from South Asia focusing on females (8–21 years). This systematic review not only contributes to identifying the trend in age at menarche and its key determinants but also highlights the regional difference and evolving research urgency in this context, thereby providing an evidence base to develop effective public health strategies and interventions.

## 2 Methods

A systematic literature analysis was undertaken to deduce the average age at menarche and to identify multiple risk factors associated with it among female adolescents in South Asian countries. The systematic review examined the sociodemographic, nutritional, lifestyle, anthropometric, biological and environmental factors associated with age at menarche among females aged 8–21 years in South Asia. The review was conducted in adherence with the Preferred Reporting Items for Systematic Reviews and Meta-Analyses (PRISMA) guidelines (34) (Supplementary Table S1—PRISMA Checklist).

The review was registered in the International Platform of Registered Systematic Review and Meta-analysis Protocols (INPLASY). (ID: INPLASY202610051).

### 2.1 Information sources

An extensive and structured literature search was carried out in four databases, including PubMed, Web of Science, Science Direct, and Google Scholar, to identify appropriate full-text research articles published in the English language across the period of 1st January 2014 and 31st May 2025. The final search was conducted on 15 July 2025. Additionally, manual screening of the reference lists of included articles was conducted to identify other eligible studies.

### 2.2 Eligibility criteria

Based on the PICOS (Population, Intervention, Comparison, Outcome, Study design, Timeframe) framework, the eligibility criteria for the review were structured to ensure clarity and consistency in the selection of studies. The PICOST criteria for the current review are presented in Table 1.

**Table 1:** Inclusion and exclusion criteria of the research articles in the present review using the PICOST framework (Population, Intervention, Comparator, Outcome, Study design, and Time frame).

Parameters	Inclusion	Exclusion
Population	Preadolescent, adolescent, and late adolescent females (8–21 years old) residing in South Asian countries, including India, Pakistan, Bangladesh, Nepal, Bhutan, Sri-Lanka, Maldives, and Afghanistan, whose age of menarche was reported	Studies involving male participants, females younger than 8 years, populations outside the 8–21-year age range, or clinical populations with specific health conditions known to significantly influence age at menarche [e.g., Turner's syndrome, polycystic ovarian syndrome (PCOS), chronic illnesses, or genetic disorders] were excluded.
Intervention	Not applicable (observational studies only)	Not applicable
Comparison	South Asian countries (Afghanistan, Bangladesh, Bhutan, India, Maldives, Nepal, Pakistan and Sri Lanka)	Countries other than South Asian countries
Outcome	Studies evaluating the factors influencing age at menarche—either contributing to early or delayed onset—or assessing determinants associated with increasing or decreasing trends in menarcheal age among adolescent females aged 8–21 years. Primary outcome: reported age at onset of menarche among pre-adolescent and adolescent females (8–21 years) in South Asia. Secondary outcomes: Factors associated with age at menarche including– sociodemographic factors (parental education and occupation, socioeconomic status, place of residence, family size, birth order); nutrition & lifestyle factors (physical activity, dietary patterns, sleep habits, stress); anthropometric factors nutritional status parameter–height, weight, BMI, waist circumference, hip circumference, waist to hip ratio, body frame assessment parameter such as bi-acromial width, bi-iliac width, and arm span; malnutrition indicators: stunting/HAZ score, wasting/WHZ scores, underweight/WAZ scores, overweight or obese (*Z* score of BMI-for-age>-2SD); biological factors (birth weight, maternal or sibling's age at menarche) and environmental factors. Trends in the age at menarche over time were also assessed.	Other than the included outcome
Study design	Observational study (cross-sectional study, case-control study, and cohort study)	Experimental study designs (randomized controlled trial and quasi-experimental design), opinion articles, and editorials
Time frame	January 2014 to May 2025	Studies published before January 2014

### 2.3 Search strategy

The search strategy utilized a combination of well-defined key words or MeSH terms as follows: [“child, female” OR “adolescents, female” OR “adolescent, female” OR “female adolescent” OR “female adolescents”] AND [“menarche” OR “age factors” OR “age at onset of menarche” OR “secular trend” OR “puberty” OR “pubertal maturation”] AND [“malnourishment” OR “malnourishments” OR “undernutrition” OR “nutritional deficiency” OR “nutritional deficiencies” OR “under-nutrition” OR “underweight” OR “stunting” OR “nutritional Status” OR “anthropometry” OR “overnutrition” OR “overweight” OR “obesity” OR “body weights and measure” OR “body mass index” OR “waist-hip ratio” OR “factor, Sociodemographic” OR “factors, sociodemographic” OR “sociodemographic Factor” OR “exposure, environ-mental” OR “environmental exposures” OR “exposures, environmental” OR “dietary Patterns” OR “dietary habits” OR “food habits” OR “habit, food” OR “eating habits” OR “eating habit” OR “habit, eating” OR “dietary habits” OR “ultra-processed foods” OR “food, ultra-Processed” OR “environmental exposure” OR “drug effect” OR “radiation exposure” OR “lifestyle factors” OR “factor, lifestyle” OR “stress, psychological” OR “physical Activity” OR “activities, physical” OR “activity, physical” OR “physical activities” OR “exercise, physical” OR “exercises, physical” OR “sedentary lifestyle” OR “life-style, sedentary” OR “physical inactivity” OR “inactivity, physical” OR “lack of physical activity”] AND [“Afghanistan” OR “Bangladesh” OR “Bhutan” OR “India” OR “Mal-dives” OR “Nepal” OR “Pakistan” OR “Sri Lanka” OR “South Asia”]. The search strategy utilized a combination of well-defined keywords and MeSH terms (where applicable) for each database, ensuring comprehensive coverage of the research question. The full search string for PubMed is provided below (Supplementary Table S2).

Similar adapted strings were used for Web of Science, ScienceDirect, and Google Scholar, taking into account database-specific syntax.

### 2.4 Study selection process

The COVIDENCE 2.0 systematic review software (Veritas Health Innovation, Melbourne, Australia) was used to import all the identified records in RIS file format from Science Direct as well as Web of Science, and in PubMed file format from the PubMed databases (35). After removing all duplicate articles, the study selection process was performed in two phases. The first step involved title and abstract screening of studies, which was undertaken independently by two reviewers. In case of any uncertainty or disagreement, the study was retained for the next phase. In the next stage, the same two reviewers independently screened the full-text research articles to identify and include the eligible studies in the review. Any discrepancies or disagreements at every stage of the review process were resolved through discussion with a third reviewer. For the review, full-text publications in the English language were included.

### 2.5 Quality assessment of selected observational studies

The methodological quality of the eligible studies was assessed using the tools developed by the Joanna Briggs Institute (JBI), Australia (36). An appropriate JBI checklist, based on study design (cross-sectional or case-control), was utilized to assess the studies (Supplementary Tables S3, S4—JBI Checklist). Two reviewers (RTW and AAM) appraised the studies independently and rated each question as “yes,” “no,” “unclear,” or “not applicable”. Any discrepancies among the two reviewers were resolved through discussion. The overall numerical score was not calculated. Based on the responses to all questions, studies were categorized as high, moderate, or low methodological quality. Studies with low quality were considered to have a high risk of bias and were interpreted cautiously (36). Given the limited availability of region-specific evidence from South Asian countries, exclusion of all studies with methodological limitations would have substantially reduced contextual representation and restricted understanding of the multifactorial determinants of age at menarche within the region. Therefore, all eligible observational studies were retained following critical appraisal using the Joanna Briggs Institute (JBI) checklist.

### 2.6 Data extraction and analysis

A systematic data extraction was conducted from all included full-text articles and organized in a Microsoft Excel spreadsheet.

Age at menarche reported is the primary outcome of this review. Therefore, any form of data related to age at menarche, including continuous variables (mean, median, standard deviations, percentiles, ranges or prevalence) or categorical variables or any specific definition (early or late) as reported, were extracted from each study and recorded in the standard format to understand the trend of age at menarche. The standardized format to record the variable is as follows: author(s), year, country, data collection method, sample size, the reported mean age at menarche, predictors of the menarcheal onset, and all broad findings. All included studies were reviewed to assess the reported age at menarche.

Further, the analysis focused on examining the association between age at menarche and different factors, which were categorized as follows:

**Sociodemographic factors:** place of residence (rural/urban), economic status, wealth index, parental educational and occupational status, birth order, caste, family size and caste/religion.

**Nutritional and Lifestyle factors:** dietary factors, including dietary habits and dietary patterns, history of complementary or breastfeeding practices; screen time, sleep, physical activity, and stress.

**Anthropometric factors:** nutritional status indicators, including undernutrition (stunting, wasting, underweight) and overnutrition (overweight and underweight, body mass index, other nutritional deficiencies, and any other anthropometric measurements such as skeletal frame, bi-acromial width, bi-iliac width, and arm span.

**Biological factors:** mother's or sister's age at menarche, birth weight.

**Environmental factors:** exposure to medicine, chemicals, or any other environmental toxins, such as household or parental use of smoking, alcohol or any other substance.

Owing to significant heterogeneity among the included studies, a meta-analysis was unfeasible; therefore, the results were narratively synthesized. A summary of findings was prepared, highlighting common patterns and associations across studies.

## 3 Results

**Figure 2:**
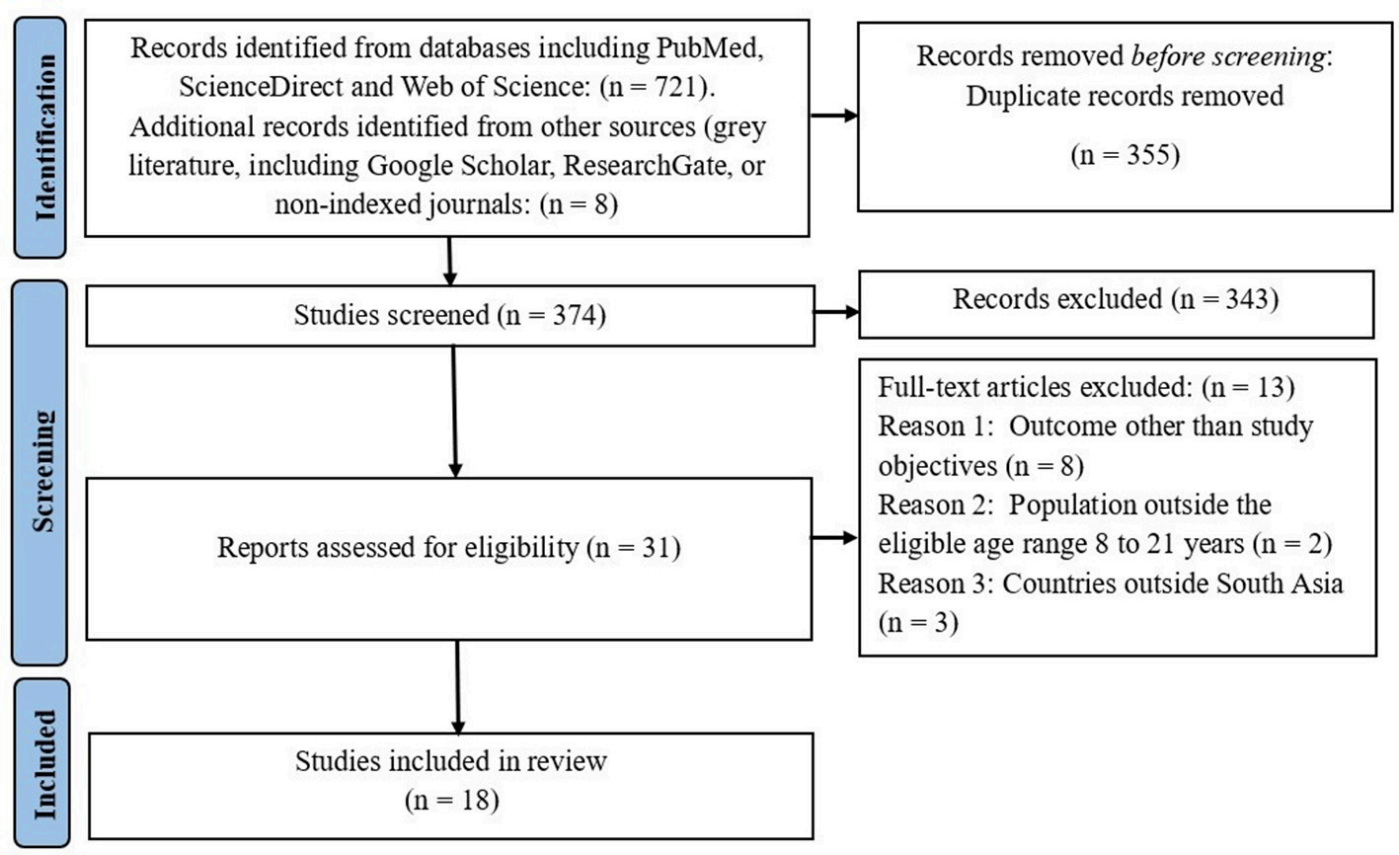
Preferred reporting items for systematic reviews and meta analysis (PRISMA) flow diagram showing the research article selection process.

An initial search in three databases (PubMed, Web of Science, and ScienceDirect) and additional searches in other sources (Google Scholar, ResearchGate, and non-indexed journals) generated 729 records. After removing 355 duplicates, the remaining 374 articles were screened based on their title and abstract. Of these, 343 articles were excluded, leaving 31 full-text articles. Out of these 31, full-text 13 studies were excluded due to reasons such as: the outcome did not match the study objectives (*n* = 8), the study population was outside the eligible age range of 8–21 years (*n* = 2), and the country was not in South Asia (*n* = 3). Consequently, 18 studies that met the eligibility criteria were included in the present systematic review. The detailed screening and selection process is depicted in the PRISMA flow diagram (Figure 2).

**Figure 3:**
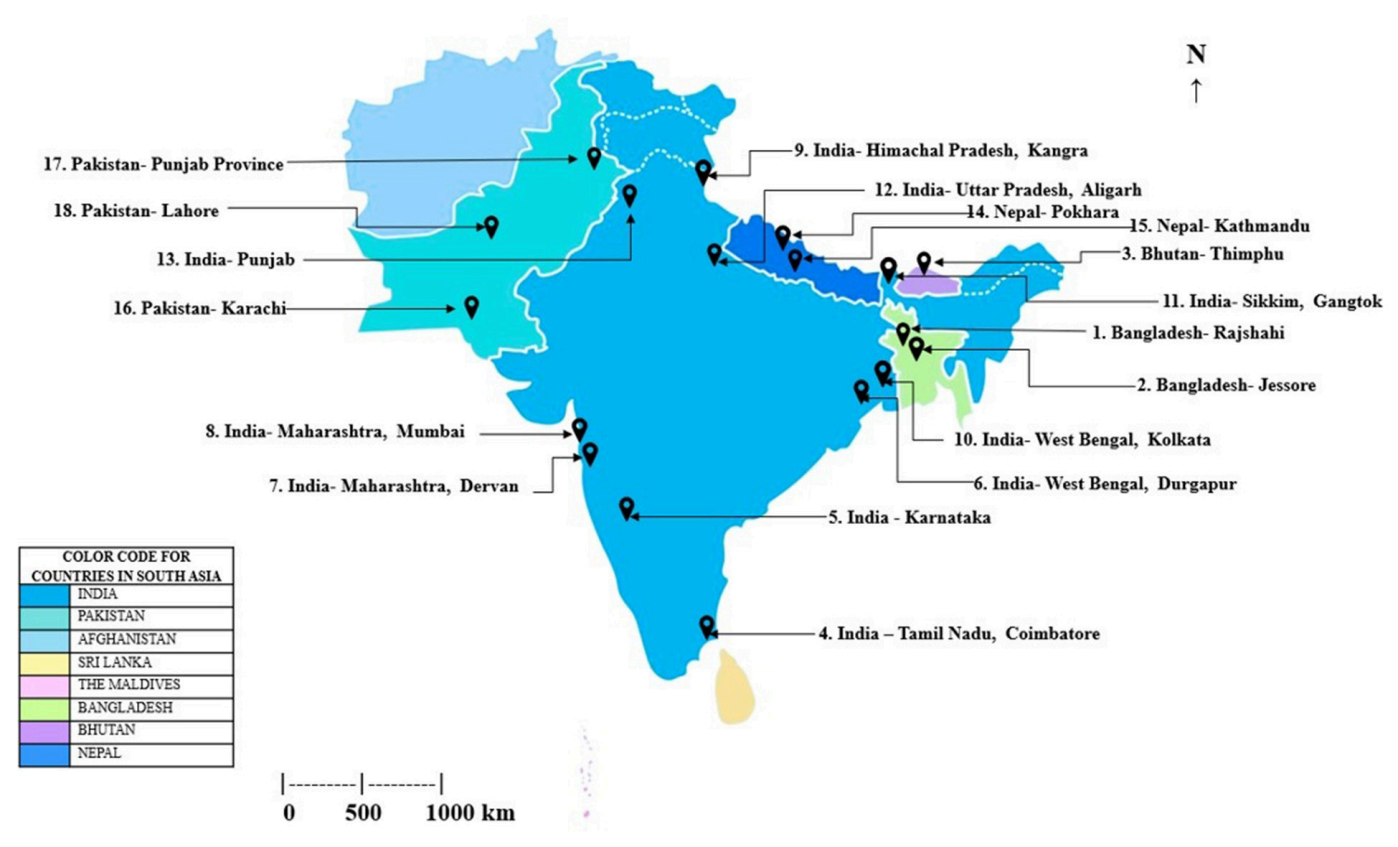
Geographic distribution of studies included in the systematic review across South Asia (*n* = 18). Study locations were color-coded by country to illustrate the geographic scope of the literature with most studies originating from India and Pakistan, and fewer studies identified from other South Asian countries.

Eighteen studies were included to prepare the manuscript. Studies originating from different countries in South Asia were included as follows: Ten from India, three from Pakistan, two from Bangladesh, two from Nepal and one from Bhutan. No studies were identified from Sri Lanka, Afghanistan and the Maldives. Figure 3 illustrates the geographical locations of the included studies.

A substantial number of records were excluded during title/abstract and full-text screening due to non-South Asian study populations, absence of extractable age at menarche data, duplicate records, inappropriate study designs, and failure to meet predefined eligibility criteria. As the review was specifically confined to South Asian countries, only a limited number of eligible studies were identified, reflecting the relative scarcity of region-specific evidence on determinants of age at menarche within South Asia.

To assess the methodological quality of 18 included studies, the Joanna Briggs Institute (JBI) critical appraisal checklists were used. Based on the response pattern, the studies were categorized as high, moderate and low quality. Three studies were rated as high quality, and six were rated as moderate quality. While nine studies were rated as low quality due to missing information on specific confounders measured or not applying an appropriate statistical method, such as multiple regression

The characteristics of the 18 included studies are presented in Supplementary Table S5. Out of the 18 studies, five [India = 2; Pakistan = 2; Nepal = 1] examined the association between sociodemographic, nutrition, lifestyle, and anthropometric factors and age at menarche (37–41). Three studies [Bangladesh = 1, India = 1, and Pakistan = 1] examined two domains, such as sociodemographic and anthropometric factors (42–44). One Indian study considered sociodemographic factors along with nutrition and lifestyle factors (45). Another single Indian study considered sociodemographic and biological factors (46). While two studies [India = 1; Bangladesh = 1] focused exclusively on sociodemographic factors (47, 48). Furthermore, two studies [India = 1; Bhutan = 1] examined four domains, including sociodemographic, nutrition and lifestyle, anthropometric and biological (49, 50). Three Indian studies have solely investigated anthropometric factors (51–53). Notably, one Nepali study included all five domains: sociodemographic, nutrition and lifestyle, anthropometric, biological, and environmental factors as predictors of age at menarche (54). The specific factor assessed under each main domain is described in Supplementary Table S5. Our findings revealed that evidence is fragmented, and a wide variation is noted among the factors studied under each main domain.

### 3.1 Trends in mean age at menarche among females from South Asian countries

Our findings indicated that most of the included studies expressed the age at menarche as mean or median. Indian studies reported the widest range (11.55–13.64 years), with most studies reporting mean ages between 12.2 and 13.5 years (37, 38, 43, 45–47, 49, 51–53). The range of mean age at menarche among Pakistani studies was narrower, which was between 11.9 and 12.6 years, suggesting a slightly earlier age at menarche when compared to Indian studies (40, 41, 44). A study from Bhutan (10.89 years) and from Bangladesh (11.6 years) also reported a slightly earlier age at menarche compared to other included studies. A Bangladeshi study did not report the mean age at menarche; however, it stated that nearly one quarter of females (25.6%) had experienced menarche by the age of 10 years (42). A case-control study from Nepal observed a noticeable difference in mean age at menarche between cases (10.8 years) and controls (14.3years) (54).

Findings revealed that the occurrence of menarche before 11 years of age and after 15 years of age is less common in South Asia. Out of 18 studies, four reported a mean age at menarche of <12 years of age (37, 43, 48, 50). Although none of the studies observed a mean age of menarche beyond 14 years of age, eight studies stated the prevalence of menarche occurring after 14 years of age, ranging from 5% to 32% (37, 44, 46, 47, 49, 51, 53). Based on these findings, establishing a standardised definition for early or late age at menarche was challenging due to substantial variations in the reported age at menarche across the included studies (Table 2).

**Table 2:** Trends and distribution of mean age at menarche among females from South Asian countries.

Sr. No.	References	Country	Mean age at menarche (years ±SD)	Sample size (*N*)	Distribution of age at menarche
1	Malitha et al. (42)	Bangladesh	NR	380	^§^10 yrs: 25.6%
					11 yrs: 41.0%
					12 yrs: 58.3%
2	Islam et al. (48)	Bangladesh	11.6 ± 3,6	24,898	8–9 yrs: 3.8%
					10–11 yrs: 40.2%
					12–13 yrs: 52.7%
					14–15 yrs: 3.4%
3	Dema and Pradhan (50)	Bhutan	10.89 ± 1.618	286	9 yrs: 5.0%
					10 yrs: 22.5%
					11 yrs: 42.5%
					12 yrs: 20.0%
					13 yrs: 6.3%
					14 yrs: 2.6%
					15 yrs: 1.3%
4	Balamurugan (37)	India	11.55 ± 1.06	790	8–10 yrs:10.8%
					11–13 yrs:72%
					14–16 yrs:17.2%
5	Sowjanya and Nagalla (38)	India	12.67 ± 1.19	700	<13 yrs: 58.9%
					>13 yrs: 41.1%
6	Agrawal and Nagalla (53)	India	13.44 ± 0.75	488	<12 yrs: 0%
					12–14 yrs: 72.95%
					>14 yrs: 27.05%
7	Patil et al. (51)	India	13.00 ± 1.1	813^a^	<12 yrs: 6.15%
					<13 yrs: 28.84%
					<14 yrs: 36.28%
					<15 yrs: 26.07%
					≥15 yrs: 6.64%
8	Dharmarha and Konda (46)	India	12.23 ± 1.09	258	9.0–9.9 yrs: 1.6%
					10.0–10.9 yrs: 5.8%
					11.0–11.9 yrs: 13.6%
					12.0–12.9 yrs: 49.6%
					13.0–13.9 yrs: 11.6%
					14 yrs: 17.8%
9	Singh et al. (45)	India	12.75 (NR SD)	276	≤ 12 yrs (Early): 41.30%
					13 yrs (Medium)^*^: 33.7%
					>14 yrs (delayed): 25%
10	Zeglen et al. (43)	India	11.8 ± 1.2	2195	Not reported
11	Pandey and Nagalla (49)	India	13.64 ± 1.58	430	10–11 yrs: 4.4%
					11–12 yrs: 17.2%
					12–13 yrs: 29.3%
					13–14 yrs: 30.2%
					14–15 yrs: 11.4%
					15–16 yrs: 5.3%
					16–17 yrs: 1.2%
					17–18 yrs: 0.9%
					18–19 yrs: 0%
12	Tarannum et al. (47)	India	12.52 ± 1.41	422	9 yrs: 0.94%
					10 yrs: 6.87%
					11 yrs: 14.92%
					12 yrs: 27.96%
					13 yrs: 24.64%
					14 yrs: 17.06%
					15 yrs: 5.45%
					16 yrs: 2.13%
13	Goyal et al. (52)	India	12.3 (NR SD)	200	11 yrs: 4%
					12 yrs: 55%
					13 yrs: 37%
					14 yrs: 4%
14	Bhattarai (54)	Nepal	Cases: 10.82 ± 0.5	Cases: 130 Control: 130	NA
			Control: 14.26 ± 0.7		
15	Chalise et al. (39)	Nepal	Mean ± SD	500	Not reported
			12.56 ± 1.12		
16	Tarar et al. (40)	Pakistan	11.93 ± 1.08	386	9 yrs: 1.2%
					10 yrs: 8.6%
					11 yrs: 19%
					12 yrs: 37.4%
					13 yrs: 22.4%
					14 yrs: 3.7%
					15 yrs: 0.9%
17	Karim et al. (44)	Pakistan	12.4	10,050	7.5–8.49 yrs: 0.01%
					8.5–9.49 yrs: 0.4%
					9.5–10.49 yrs: 2.4%
					10.5–11.49 yrs: 7.9%
					11.5–12.49 yrs: 20.8%
					12.5–13.49 yrs: 17.9%
					13.5–14.49 yrs: 7.1%
					14.5–15.49 yrs: 1.8%
					15.5–16.49 yrs: 0.4%
18	Khalid et al. (41)	Pakistan	Mean ± SD	199	Simple distribution: the overall percentage by age is not reported
			12.66 ± 1.12		

*N*, sample size; NR, not reported; NA, not applicable; SD, standard deviations; yrs, years; a, sample size for Patil et al. refers to post-menarcheal girls (813 out of 1,071 total); NR SD, SD is not reported; ^§^The total sum reported for percentage categories at distribution of age at menarche by Malitha et al. is >100%. The data is presented as reported by the author; In the study by Patil et al., age categories of the distribution of age at menarche percentages are reported in cumulative form. Data is presented as reported by the author.

Thus, to synthesize the review, an operational definition was adopted: menarche occurring before 12 years was categorised as early and after the age of 14 years was categorised as late.

### 3.2 Sociodemographic factors and age at menarche

Among sociodemographic factors, place of residence, parental educational and occupational status, economic status, caste, and religion were identified as determinants of age at menarche.

A Bangladeshi study reported a higher risk of early menarche in females residing in urban areas compared to females from rural areas (Rural vs. Urban: AOR = 0.012, 95% CI: 0.003–0.047; *p* < 0.01). However, this study did not report the mean age at menarche for either group, thereby limiting the comparison of the actual age at menarche between both groups (42). Similarly, an Indian study observed an earlier age at menarche among females residing in urban areas than their rural counterparts (Area of residence: β coefficient = 0.264; 95% CI: −0.955, −0.552); *p* = 0.001 (37). Another Bangladeshi study reported that females who studied till higher grade had earlier menarche (AOR = 2.74; 95% CI: 1 (1.899–3.955); *p* < 0.0001) (48).

Further, the influence of parental traits on age at menarche was demonstrated by a few studies. For instance, a Nepali study found that the absence of a biological father increased the risk of early menarche (AOR = 10.001, 95% CI: 1.195–83.660; *p* = 0.034 (54). Likewise, a Pakistani study reported that girls whose fathers worked in the private sector menarche earlier than those whose fathers worked in government or agriculture, but these findings are not reliable as they were derived from a low-quality study (40).

With regards to socioeconomic class, an Indian study reported a higher prevalence of early menarche in females from higher socioeconomic class (35.9%) as compared to females from middle (3.62%) and lower socioeconomic (1.81%) classes, and the difference was statistically significant (χ^2^ = 31.467, *p* < 0.01) (45). This is supported by another Indian study that found 23.2% of girls from the higher socioeconomic class experienced menarche before the age of 12, as compared to girls from the lower class (5.9%) (46). However, both these studies were identified as low quality. Conversely, a Pakistani study reported late age at menarche in girls belonging to lower socioeconomic status than those belonging to middle/high socioeconomic class (44). On the contrary, an Indian study reported an early age at menarche in females from lower socioeconomic class (49). While a Nepali study stated no association between age at menarche with family income, caste or race (39). Overall, regarding the association between socioeconomic status and age at menarche, diverse findings may be attributed to variation in context and differences in the quality of studies. Interestingly, socioeconomic status indicators such as more rooms (living space) and access to improved toilet facilities were linked to early age at menarche by one Indian study (43).

Additionally, a study found a significant association between birth order and age at menarche (*F* = 8.485; *p* < 0.0001). The study noted a lower mean menarcheal age in girls who were born first or second (12.21 ± 1.35 years) than those born third or fourth (12.77 ± 1.26 years) or later (12.79 ± 1.79 years). These findings cannot be interpreted casually, as this study was of low quality due to failure in controlling potential variables, including socioeconomic status, nutritional status and maternal age at menarche (47).

These discrepancies may be attributed to regional or cultural differences. In essence, the variations noted about the relationship between menarcheal age and sociodemographic characteristics in South Asian contexts not only reflect the intricate interactions between urbanization, living conditions, and nutritional status but also differences in study methodologies.

### 3.3 Nutrition and lifestyle factors and age at menarche

Under this domain, variables including diet, food habits, screen time, physical activity, and sleep duration have been extensively investigated.

#### 3.3.1 Nutrition

Dietary patterns significantly influence the timing of menarche. A study reporting early age at menarche in girls who consumed fruits and vegetables frequently, indicating a possible link between improved dietary quality and earlier pubertal onset (37). However, findings regarding dietary types are not consistent in all studies. One Indian study revealed that adherence to a non-vegetarian diet increased the risk of early menarche among females (38). Whereas a Nepali study revealed no significant association between dietary habits (vegetarian vs. non-vegetarian) and age at menarche (39). These conflicting results may suggest that the relationship between diet type and menarcheal timing may differ based on dietary composition, regional patterns, and nutritional quality. Early life nutrition is also found to be important in determining the age at menarche. One study found that inadequate breastfeeding was associated with a higher likelihood of early menarche (AOR = 5.204; 95% CI: 1.544–17.543; *p* = 0.001), indicating that suboptimal infant nutrition may accelerate menarcheal timings (54).

#### 3.3.2 Lifestyle factors (physical activity, sleep and screen time)

Physical activity, sleep, and media exposure were also identified as key determinants of age at menarche. Excessive exposure to audio-visual media and late sleeping habits were identified as risk factors for early menarche (38). Interestingly, one Indian study reported that physically more active females attained menarche at an earlier age compared to their less active counterparts, but this study was rated as low quality (45). On the contrary, a Nepali study reported that physical inactivity (AOR = 5.694; 95% CI: 1.932–16.787; *p* < 0.001) and insufficient sleep (AOR = 8.077; 95% CI: 2.628–24.830; *p* = 0.001) increased the odds of early menarche (54). Conversely, a Pakistani study reported no significant association between age at menarche and physical activity or stress levels (41).

The divergent findings may be attributed to variations in study methodologies, such as recall bias, different tools to assess diet or physical activity and socioeconomic status, and statistical methods applied. In addition, contextual factors such as diet, body composition, cultural practices and urban lifestyle patterns may influence the association between physical activity and menarcheal timing.

#### 3.3.3 Anthropometric factors and age at menarche

Ten of the 18 studies examined the relationship between body mass index (BMI) and menarcheal age, indicating that it was the most extensively researched determinant among all the anthropometric variables. A higher BMI has been repeatedly linked to an earlier menarche (38, 40–44, 49–51, 53) implying that increased adiposity may hasten pubertal development. However, significant differences in methodological qualities were noted among the studies that demonstrated these associations. Two studies were of low quality due to flaws in methodology, such as the absence of multivariate analysis; therefore, inferences from these studies should be drawn cautiously (40, 50). Conversely, two studies that found no association between age at menarche and BMI were also of low quality (41, 53). Remarkably, methodologically robust studies revealed that higher BMI is an independent predictor of early age at menarche.

An Indian study which investigated the relationship of age at menarche with stunting and thinness found that the risk of thinness increased with later menarche compared to the girls with age at menarche <12 years of age; <13 years (OR = 3.65, 95% CI: 1.08–12.4, *p* < 0.001); <14 years (OR = 7.25, 95% CI: 2.20–23.89, *p* < 0.001); <15 years (OR = 10.69, 95% CI: 3.22–35.46*, p* = 0.001); and ≥15 years (OR = 12.43,95% CI: 3.42–45.1, *p* = 0.04). Contrary to this, lower risk of overweight was observed in girls with higher age at menarche, for example, <14years (OR = 0 13, 95% CI: 0.04–0.43, *p* = 0.001); <15 years (OR = 0.29, 95% CI: 0.09–0.87, *p* = 0.027). A similar trend was observed for stunting; however, a statistically significant association was observed for the higher group only (≥15years: OR=3.43, 95% CI: 1.29–9.11, *p* = 0.013) (51). Additionally, these findings are supported by the study, which applied continuous height measure in analysis and found that the probability of earlier age at menarche was 70% in females whose height-for-age *Z*-score (HAZ) was two (43). Overall, findings indicate that undernourished females have a higher risk of delayed menarche compared to their well-nourished counterparts.

Additionally, an Indian study evaluated the relationship between menarcheal age and other anthropometric parameters, including body weight, height, biacromial width, bi-iliac width, and arm span. No significant correlation was found between age at menarche and body weight (*r* = −0.073; *p* = 0.122) or bi-iliac width (*r* = 0.252; *p* = 0.024). However, height (*r* = 0.170; *p* = 0.003), biacromial width (*r* = 0.310; *p* = 0.048), and arm span (*r* = 0.198; *p* = 0.009) showed significant positive correlations, indicating that girls with greater linear growth and skeletal breadth tended to attain menarche later (52).

Overall, these findings highlight that both overnutrition (higher BMI) and undernutrition (stunting, thinness) influence the timing of menarche, while other anthropometric characteristics related to growth and skeletal development also play a significant role in the onset of menarche among South Asian girls. A study conducted in Bhutan reported a significant association between age at menarche and BMI. However, the overall findings of this study remain unclear, as the author didn't explicitly mention the list of variables adjusted for in the regression model (50).

### 3.4 Biological factors and age at menarche

Family history (age at menarche of female relatives) was found to influence the timing of menarche. In one study, sisters' age at menarche was distributed as follows: 28.1% at 10–12years, 50.4% at 12–14 years, and 21.5% at 14–16 years (*p* = 0.002), suggesting a significant familial pattern in menarcheal timing (46). Similarly, another study found that a mother's age at menarche was also strongly associated with the daughter's menarcheal age (*r* = 0.549; *p* < 0.001). For example, among daughters whose mothers attained menarche between 11 and 15 years, 100% experienced menarche at 10–11 years and 89.2% at 12–14 years. In contrast, daughters of mothers with menarche at 16–20 years were more likely to experience menarche at 12–14 years (10.2%) or ≥15years (75%). However, this study was rated as low quality due to not controlling for potential confounders such as BMI and socioeconomic status. Although the author explicitly mentioned descriptive data, the association found should be interpreted cautiously (49). Moreover, early life factors were identified as a key predictor. Girls with low birth weight had nine times higher odds of early menarche compared to those with normal birth weight (AOR = 9.444; 95% CI: 3.022–29.517; *p* = 0.002) (54). These findings indicated that prenatal growth, early-life nutritional status and familial factors are the key biological factors influencing age at menarche.

### 3.5 Environmental factors and age at menarche

Out of 18 studies, only one examined the association between environmental factors and menarcheal timing. This study revealed that exposure to sexual material (AOR = 34.782; 95% CI: 6.366–190.026; *p* = 0.008), maternal use of hormonal contraceptives during pregnancy (AOR = 5.805; 95% CI: 1.722–19.568; *p* = 0.005), and mothers' prenatal exposure to chemicals (AOR = 3.917; 95% CI: 1.159–13.234; *p* = 0.028) increased risk of early menarche in daughters. These findings highlight the potential influence of in-utero and environmental exposures on pubertal development and timings of menarche (54). The factors associated with age at menarche are summarized in Supplementary Table S6.

Based on the findings from only moderate to high quality studies the review summarizes that the menarche occurring before the age of 12 was commonly associated with associated with urban residence, a higher standard of living, higher BMI, inadequate sleep and physical activity, higher consumption of fruits, vegetable and non-vegetarian diet, excessive screen time, low birth weight, absence of biological father, mothers' age at menarche and exposure to environmental factors. In contrast, the menarche occurring after the age of 14 years was associated with factors such as rural residence, lower socioeconomic status, and indicators of undernutrition, including stunting and underweight (Table 3).

**Table 3:** Summary of factors associated with age at menarche among females aged 8–21 years in South Asia.

Factors	Factors associated with age at menarche occurring before the age of 12	Factors associated with age at menarche occurring after 14 years of age
Sociodemographic factors	Urban residence (37, 42)	Rural residence (51)
	Higher education of female adolescents (48)	Lower socioeconomic status (44)
	Higher socioeconomic status (45, 48)	
	Higher standard of living (43)	
	Higher occupational status of father (40)	
	Absence of biological father (54)	
Nutrition and lifestyle factors	High frequency of consumption of fruits and vegetables (37)	–
	Non-vegetarian (38)	
	Inadequate breastfeeding (54)	
	Inadequate physical activity (38, 54)	
	Adequate physical activity (45)	
	Excessive screen time/exposure to audio-visual media (38) Inadequate sleep (38, 54)	
Anthropometric factors	High BMI (38, 40, 42–44, 49, 53)	Stunting, underweight (51)
Biological factors	Low birth weight (54) Maternal age at menarche (49, 54)	–
Environmental factors	Exposure to sexual material, use of hormonal contraceptives by the mother, exposure to chemicals during pregnancy (54)	–

BMI, body mass index.

## 4 Discussion

This systematic review encompassing 18 selected research articles critically examined the association between sociodemographic, nutritional, lifestyle, anthropometric, biological and environmental factors and the age at menarche among females aged 8–21 years in the underexplored South Asian region.

The mean age at menarche across most South Asian studies lies within 12–13 years, aligning well with global averages (2). However, the reported mean age of menarche in studies from Bhutan, Bangladesh and Nepal was under 12 years, whereas the prevalence of late menarche after 14 years of age was reported in some Indian studies, indicating significant sub-regional variation in mean age at menarche (39, 42, 48, 50, 54).

Menarche occurring before the age of 12 years was consistently associated with sociodemographic factors such as urban residence, and a higher standard of living; lifestyle factors, including higher BMI, sleep, and physical activity (38, 40, 42–44, 49), higher consumption of fruits and vegetables and non-vegetarian diet, excessive screen time, and biological/environmental factors like low birth weight, absence of biological father, mothers' age at menarche, and exposure to environmental factors. Conversely, menarche occurring after 14 years of age was associated with rural residence, lower socioeconomic status, and undernutrition (stunting and underweight) (37, 38, 40, 41, 51, 54).

Indian studies demonstrated the association between sociodemographic factors, particularly place of residence and age at menarche (37, 47, 51). A Bangladeshi study, which applied an advanced statistical model adjusting for potential confounders (BMI category, monthly family income, maternal age and stature, parental occupation and education, mode of birth, type of residence), revealed that the odds of early age at menarche were higher in girls living in urban areas (48). Similarly, an Indian study conducted in a similar setting also reported early age at menarche in females residing in urban areas (37). Conversely, another Indian study conducted in a rural setting reported that almost 32% female experienced menarche after 14 years of age (51). Several other studies from various other nations also reported the urban-rural disparity in age at menarche (55–57). Besides regional disparity, socioeconomic disparity is also linked with age at menarche. For instance, a Pakistani study found delayed age at menarche in girls belonging to the lower socioeconomic backgrounds (44). Conversely, another Indian study demonstrated that the association of a high standard of living, reflected in household possessions including additional room and toilet facility, with an earlier age at menarche (43). These findings are consistent with a study conducted in China, which reported that better economic conditions and standard of living are linked with earlier age at menarche (58). Although there is evidence that sociodemographic and regional factors are linked with the age at menarche, these linkages are ultimately influenced by the proximal factors such as nutritional status, lifestyle, psychological and environmental factors (59).

In addition to sociodemographic disparities, nutritional disparities are also key determinants of age at menarche. Our review notably found an Indian study showing a counterintuitive correlation between eating more fruits and vegetables and the early occurrence of menarche, consistent with higher consumption of non-vegetarian food also linked with early age at menarche (37, 38). Although these results may seem paradoxical, they are not a direct biological consequence of eating more vegetables, fruits, or non-vegetarian food but rather a reflection of broader nutritional and socioeconomic settings. Similar results were observed across the world (1, 16). Thus, these associations suggest that both early and late age at menarche are consequences of total dietary calorie accessibility and availability rather than consumption of specific food items.

At the other end, nutritional deficiency leads to delayed menarche. An Indian study demonstrated a strong correlation between delayed menarche, energy deficiency and compromised nutritional status (51). Variations in diet-related findings may be due to differences in the methodologies or tools used to assess dietary data across studies. Studies from sub-Saharan Africa and East Asia reported stunting, wasting and delayed age at menarche among undernourished populations (60). The intensity of physical activity has also shown a positive correlation with delayed menarche, as evidenced in a global systematic review (14). On the contrary, studies from Nigeria and China reveal that early menarche patterns have been strongly associated with energy-dense dietary patterns and sedentary pursuits (7, 61–63).

Apart from these lifestyle and behavioural factors, psychosocial factors such as the absence of a biological father were identified as a predictor of early menarche. This result aligns with other international studies, which documented linkages between parental absence, family structure with onset of puberty, driven by psychosocial stress (64). These findings are in line with global evidence, suggesting that higher BMI with increased adiposity and altered hormonal balance are the key drivers of declining age at menarche. This calorie surplus is largely attributed to energy-dense, empty-calorie, poor dietary habits, sedentary lifestyle, and poor sleep patterns (11, 65). Biologically, increased body fat signals activation of the hypothalamic-pituitary-gonadal axis, leading to increased synthesis of leptin and estrogen hormones, eventually hastening the onset of puberty (66). This mechanism has been observed in Western and East Asian cohorts (58, 67, 68). The present review findings for South Asia are consistent with the worldwide evidence (37, 38, 54).

Early life nutrition and lifestyle programming are evidence-based biological factors shaping menarche timing and predicting future disease risks. A worldwide meta-analysis ascertained similar results, where exclusive breastfeeding for every extra month could delay the menarche by 0.31 months (69). The strong correlation between the menarcheal ages of the mother and her siblings suggests a significant genetic component, which is in line with findings from throughout the world that genetics is mostly responsible for the diversity in age of menarcheal onset (16, 42, 46, 49, 54).

Elaborating on these early life factors, the prenatal environment is instrumental in shaping pubertal trajectories. Maternal nutritional status has been the most significant factor correlated with the age at menarche of girls. Extreme situations, such as poor weight gain (<10 lbs) or excessive weight gain (>40 lbs), are associated with an earlier age at menarche in children (14). Likewise, low birth weight was identified as a key determinant of early age at menarche, indicating that postnatal catch-up growth and intrauterine undernutrition may modify hormone regulation through epigenetic processes (70).

Beyond prenatal environment and early development, environmental exposures during early childhood also impact age at menarche. Early exposure to sexual material, prenatal chemical exposure, and maternal hormonal usage were all linked to an earlier menarche. This is consistent with studies across the globe suggesting widespread exposure to hormones and endocrine disruptors, such as passive and active smoke, might hasten the beginning of puberty (15, 54, 71). These observations reinforce the necessity for focused studies on the prenatal and environmental determinants that influence menarche in the South Asian populations.

While BMI is often considered the most established variable to evaluate preadolescent health trajectories, the current review found that most included studies were limited to BMI (45, 53, 55). A very few studies explored other measures such as height, arm-span, shoulder width or other body measurements, which also change with pubertal growth (52). As highlighted in a Lancet report, it is important for future research to critically examine how BMI interacts with environmental factors such as sedentary lifestyle, dietary patterns, and psychosocial stressors, as these can influence menarcheal timings (27).

### 4.1 Strengths and limitations

This is the first systematic review to comprehensively examine a wide range of socio-demographic, anthropometric, nutritional, lifestyle, biological and environmental factors associated with the age at menarche across South Asia. The key strength of this review lies in its robust methodological approach, which included extensive search strategies across four major databases and adherence to JBI guidelines, ensuring a systematic and unbiased study selection process. Furthermore, the included studies have larger sample sizes, and most of them used robust methodologies to identify the factors associated with age at menarche.

However, the present review is limited by reliance only on studies published in the English language. Recall bias in reporting the age at menarche, a limitation clearly stated in several included studies, may lead to imprecision in the reported mean age and association within our synthesis. The review noted that none of the 18 included studies clearly defined early or late menarcheal age, which restricted the comparison of the findings across studies. The certainty of our findings was impacted due to the inclusion of some low methodological quality studies. A formal sensitivity analysis excluding low-quality studies was not feasible due to the small number of eligible studies (*n* = 18). While narrating the findings, we have presented a cautious interpretation of them. Additionally, due to the heterogeneity of statistical methods and the limited use of advanced analytical statistical tests, the extent to which causal inferences can be drawn. Importantly, the relatively small number of studies identified across the South Asian region appears to reflect a genuine scarcity of rigorous and region-specific research on determinants of age at menarche rather than overly restrictive inclusion criteria. Despite comprehensive database searching, no eligible studies were identified from Maldives, Sri Lanka, or Afghanistan, and the available evidence was disproportionately concentrated in India. Consequently, the findings may not fully represent the sociocultural, nutritional, environmental, and demographic diversity of the broader South Asian population, thereby limiting the regional generalisability of the conclusions. The synthesis should be a viewed as a summary of available evidence from a few settings, not as a definitive characterization of entire region.

### 4.2 Future research directions

In the preadolescent stage itself, health, education-oriented, and nutritional interventions should be initiated to attenuate the future maternal and neonatal complications as well as enhance the future pregnancy health outcomes, thereby adding to a wider range of health and economic benefits. Although pre-conception health may not be the key focus of the health interventions, aiming to nurture the reproductive system's health right from early adolescence will mitigate the future complications and achieve a better reproductive health (17).

Extremes of menarcheal timings are linked with adverse health outcomes such as early sexual debut and substance use in adolescents. This evidence is limited to high-income countries due to a paucity of data for low- and middle-income countries. Age at menarche, being a crucial indicator for the nutritional status and future reproductive health of females, has been suggested that data on average age at menarche needs to be systematically collected in national Demographic and Health Surveys (DHS) and country-level surveys (72). This would be essential for the nutritionist and reproductive health expert for public health planning. To our knowledge, this data is not yet incorporated in the current national health surveillance systems in South Asia. The present review also noted that none of the included studies could clearly define or report a population-based average for early menarche, underlining this vital measurement gap.

In such a context, the Global Accelerated Actions—Health of Adolescents (AA-HA) framework of WHO provides a life course approach highlighting the role of policies and socioeconomic conditions, media exposure and access to supportive environments during the growing period of female adolescents, which determines their nutritional status, and potential implications on menarcheal timings (21).

Future research should prioritize well-designed, region-specific, and longitudinal studies in South Asia. For cross-study comparison, a consistent definition of early and late menarche and diverse anthropometric parameters in addition to BMI are needed. Additionally, priority to be given to incorporating age at menarche into national surveillance data.

The review's findings highlight that the age at menarche is a crucial biological milestone, shaped by a complex interplay of socioeconomic, nutritional, lifestyle, anthropometric, biological and environmental factors rather than any single determinant as depicted in the conceptual framework (Figure 1). Despite the limitations, this review is the first to fundamentally map the evidence on the multifactorial determinants of age at menarche in South Asia. A foundation for future research and development for effective public health strategies and a need for targeted interventions can be laid through this review.

## 5 Conclusion

This review confirms that the multifactorial influences on age at menarche in South Asia, with early menarche, were associated with markers of urbanisation, including urban residence, a higher standard of living, lifestyle factors (e.g., higher BMI, inadequate sleep and physical activity, screen time, and particular dietary pattern) and specific biological and environmental factors. In contrast, late menarche was consistently associated with rural residence, lower socioeconomic status, and undernutrition. These findings have substantial health consequences for adolescent females.

The mixed results suggest the role of regional, cultural, contextual, and methodological differences across studies. Importantly, biological factors like maternal nutritional status, early nutrition and birth weight, though non-modifiable for the individual, are crucial in the growth and development trajectories in future. Thus, an intergenerational approach to optimise the age at menarche should be highlighted.

By addressing the modifiable factors such as dietary patterns, physical activity, stress and lifestyle, the timing of menarche can be modulated to occur within a normal age-appropriate range. By consolidating the evidence on menarcheal timing and its determinants, this review generates region-specific insights essential for informing and adapting the public health and nutritional interventions for better health outcomes in adolescence and womanhood.

## Data Availability

The original contributions presented in the study are included in the article/Supplementary material, further inquiries can be directed to the corresponding author.
