# Supplementary material for: Age at menarche in South Asia: an interplay of sociodemographic, nutritional, lifestyle, anthropometric, biological, and environmental factors—a systematic review
Source: Front Public Health. 2026 Jul 15;14:1836422. doi: 10.3389/fpubh.2026.1836422 (PMC13415688; doi:10.3389/fpubh.2026.1836422)
Supplement: Supplementary file 1 [file Table_1.docx]

**SUPPLEMENTARY MATERIAL**

**Table S1:** PRISMA Checklist

| **Section and Topic** | **Item #** | **Checklist item** | **Location where item is reported** |
| --- | --- | --- | --- |
| **TITLE** | | |  |
| Title | 1 | **Identify the report as a systematic review:**  Age at Menarche in South Asia: An Interplay of Sociodemographic, Nutritional, Lifestyle, Anthropometric, Biological and Environmental Factors – A Systematic Review | Title page |
| **ABSTRACT** | | |  |
| Abstract | 2 | **See the PRISMA 2020 for Abstracts checklist:** | Abstract |
| **INTRODUCTION** | | |  |
| Rationale | 3 | **Describe the rationale for the review in the context of existing knowledge:** | Introduction |
| Objectives | 4 | **Provide an explicit statement of the objective(s) or question(s) the review addresses:** | Introduction |
| **METHODS** | | |  |
| Eligibility criteria | 5 | **Specify the inclusion and exclusion criteria for the review and how studies were grouped for the syntheses.** | 2.2 Eligibility criteria |
| Information sources | 6 | **Specify all databases, registers, websites, organizations, reference lists and other sources searched or consulted to identify studies. Specify the date when each source was last searched or consulted:** | 2.1 Information  sources |
| Search strategy | 7 | **Present the full search strategies for all databases, registers and websites, including any filters and limits used:** | 2.3 Search Strategy |
| Selection process | 8 | **Specify the methods used to decide whether a study met the inclusion criteria of the review, including how many reviewers screened each record and each report retrieved, whether they worked independently, and if applicable, details of automation tools used in the process.**  . | 2.4  Study Selection Process |
| Data collection process | 9 | **Specify the methods used to collect data from reports, including how many reviewers collected data from each report, whether they worked independently, any processes for obtaining or confirming data from study investigators, and if applicable, details of automation tools used in the process.** | 2.5Quality assessment of selected observational studies |
| Data items | 10a | List and define all outcomes for which data were sought. Specify whether all results that were compatible with each outcome domain in each study were sought (e.g. for all measures, time points, analyses), and if not, the methods used to decide which results to collect. | 2.6  Data extraction and analysis |
|  | 10b | **List and define all other variables for which data were sought (e.g. participant and intervention characteristics, funding sources). Describe any assumptions made about any missing or unclear information** | 2.6  Data extraction and analysis  Funding |
| Study risk of bias assessment | 11 | **Specify the methods used to assess risk of bias in the included studies, including details of the tool(s) used, how many reviewers assessed each study and whether they worked independently, and if applicable, details of automation tools used in the process.** | Methods |
| Effect measures | 12 | **Specify for each outcome the effect measure(s) (e.g. risk ratio, mean difference) used in the synthesis or presentation of results:** | NA |
| Synthesis methods | 13a | **Describe the processes used to decide which studies were eligible for each synthesis (e.g. tabulating the study intervention characteristics and comparing against the planned groups for each synthesis (item #5)):** | Table 1 |
|  | 13b | **Describe any methods required to prepare the data for presentation or synthesis, such as handling of missing summary statistics or data conversions:** | NA |
|  | 13c | **Describe any methods used to tabulate or visually display the results of individual studies and syntheses:** | NA |
|  | 13d | **Describe any methods used to synthesize results and provide a rationale for the choice(s). If meta-analysis was performed, describe the model(s), method(s) to identify the presence and extent of statistical heterogeneity, and software package(s) used:** | NA |
|  | 13e | **Describe any methods used to explore possible causes of heterogeneity among study results (e.g. subgroup analysis, meta-regression):** | NA |
|  | 13f | **Describe any sensitivity analyses conducted to assess robustness of the synthesized results:** | NA |
| Reporting bias assessment | 14 | **Describe any methods used to assess risk of bias due to missing results in a synthesis (arising from reporting biases):** | NA |
| Certainty assessment | 15 | **Describe any methods used to assess certainty (or confidence) in the body of evidence for an outcome:** | NA |
| **RESULTS** | | |  |
| Study selection | 16a | **Describe the results of the search and selection process, from the number of records identified in the search to the number of studies included in the review, ideally using a flow diagram:** Figure 2: PRISMA flow diagram | Results |
|  | 16b | Cite studies that might appear to meet the inclusion criteria, but which were excluded, and explain why they were excluded.  The following studies were excluded because of the wrong region (n=3)  **[1]** Chen, Y.; Liu, J. Do most 7- to 8-year-old girls with early puberty require extensive investigation and treatment? *Journal of Pediatric and Adolescent Gynecology* **2021**, *34*, 124–129. <https://doi.org/10.1016/j.jpag.2020.11.020>  **[2]** Xu, H.; Wen, Q.; Xing, X.; Chen, Y.; Zhu, Q.; Tan, M.; Zhang, M.; Pan, T.; Wu, S. High dietary inflammatory index increases the risk of female infertility: An analysis of NHANES 2013–2018. *Nutrition Research* **2024**, *125*, 50–60. <https://doi.org/10.1016/j.nutres.2024.02.006>  **[3]** Yu, E.J.; Choe, S.-A.; Yun, J.-W.; Son, M. Association of early menarche with adolescent health in the setting of rapidly decreasing age at menarche. *Journal of Pediatric and Adolescent Gynecology* **2020**, *33*, 264–270. <https://doi.org/10.1016/j.jpag.2019.12.006>  The following studies were excluded because of the wrong outcomes (n=10)  **[1]** Borle, A.L.; Gangadharan, N.; Basu, S. Lifestyle practices predisposing adolescents to non-communicable diseases in Delhi. *Dialogues in Health* **2022**, *1*, 100064. <https://doi.org/10.1016/j.dialog.2022.100064>  **[2]** Burris, M.E.; Wiley, A.S. Marginal food security predicts earlier age at menarche among girls from the 2009–2014 National Health and Nutrition Examination Surveys. *Journal of Pediatric and Adolescent Gynecology* **2021**, *34*, 462–470. https://doi.org/10.1016/j.jpag.2021.03.010  **[3]** Ganie, M.A.; Chowdhury, S.; Suri, V.; Joshi, B.; Bhattacharya, P.K.; Agrawal, S.; Malhotra, N.; Sahay, R.; Jabbar, P.K.; Nair, A.; *et al.* Normative range of various serum hormonal parameters among Indian women of reproductive age: ICMR-PCOS task force study outcome. *The Lancet Regional Health – Southeast Asia* **2023**, *15*, 100226. https://doi.org/10.1016/j.lansea.2023.100226  **[4]** Magalhães, A.C.L.; Pierucci, A.P.; Oliveira, M.N.; Campos, A.B.F.; Jesus, P.C.; Ramalho, A. Relationship of age at menarche and serum leptin with the metabolically unhealthy phenotype in adolescents. *Nutrición Hospitalaria* **2021**, *38*, 29–35. https://doi.org/10.20960/nh.03050  **[5]** Mathad, V.; Badiger, S.; Manjunath, N. Assessment of anemia and malnutrition among adolescents in the Kalyan Karnataka region of Karnataka. *Clinical Epidemiology and Global Health* **2023**, *21*, 101307. https://doi.org/10.1016/j.cegh.2023.101307  **[6]** Nasiri, S.; Dolatian, M.; Ramezani Tehrani, F.; Alavi Majd, H.; Bagheri, A. The relationship between social determinants of health and girls’ age at menarche based on the World Health Organization model: Path analysis. *Heliyon* **2022**, *8*, e10794. https://doi.org/10.1016/j.heliyon.2022.e10794  **[7]** Ranasinghe, C.; Shettigar, P.G.; Garg, M. Impact of parity and breast-feeding duration on body mass index among post-menopausal women. *Clinical Epidemiology and Global Health* **2019**, *7*, 626–628. https://doi.org/10.1016/j.cegh.2019.02.005  **[8]** Sharma, P.; Kaur, M.; Kumar, S.; Khetarpal, P. A cross-sectional study on prevalence of menstrual problems, lifestyle, mental health, and PCOS awareness among rural and urban populations of Punjab, India. *Psychosomatic Obstetrics & Gynecology* **2022**, *43*, 349–358. https://doi.org/10.1080/0167482X.2021.1965983  **[9]** Upadhyay, M.; Mahishale, A.; Kari, A. Prevalence of premenstrual syndrome in college-going girls: A cross-sectional study. *Clinical Epidemiology and Global Health* **2023**, *20*, 101234. https://doi.org/10.1016/j.cegh.2023.101234  **[10]** Vijayakumar, N.; Youssef, G.; Bereznicki, H.; Dehestani, N.; Silk, T.J.; Whittle, S. The social determinants of emotional and behavioral problems in adolescents experiencing early puberty. *Journal of Adolescent Health* **2024**, *74*, 674–681. <https://doi.org/10.1016/j.jadohealth.2023.06.025> |  |
| Study characteristics | 17 | **Cite each included study and present its characteristics:**  **Table S4.** Characteristics of the included research articles assessing the factors associated with age at menarche among females in studies selected from South Asian countries. | Table S4 |
| Risk of bias in studies | 18 | **Present assessments of risk of bias for each included study:** | NA |
| Results of individual studies | 19 | **For all outcomes, present, for each study: (a) summary statistics for each group (where appropriate) and (b) an effect estimate and its precision (e.g. confidence/credible interval), ideally using structured tables or plots:**  NA | NA |
| Results of syntheses | 20a | **For each synthesis, briefly summarise the characteristics and risk of bias among contributing studies:**  Table S2, Table S3, Table S4 | Table S2  Table S3  Table S4 |
|  | 20b | **Present results of all statistical syntheses conducted. If meta-analysis was done, present for each the summary estimate and its precision (e.g. confidence/credible interval) and measures of statistical heterogeneity. If comparing groups, describe the direction of the effect**.  NA | NA |
|  | 20c | **Present results of all investigations of possible causes of heterogeneity among study results.**  NA | NA |
|  | 20d | **Present results of all sensitivity analyses conducted to assess the robustness of the synthesized results.**  NA | NA |
| Reporting biases | 21 | **Present assessments of risk of bias due to missing results (arising from reporting biases) for each synthesis assessed.**  **NA** | NA |
| Certainty of evidence | 22 | **Present assessments of certainty (or confidence) in the body of evidence for each outcome assessed.** | NA |
| **DISCUSSION** | | |  |
| Discussion | 23a | **Provide a general interpretation of the results in the context of other evidence:** | Discussion |
|  | 23b | **Discuss any limitations of the evidence included in the review:** | Discussion |
|  | 23c | **Discuss any limitations of the review processes used:** | Discussion |
|  | 23d | **Discuss implications of the results for practice, policy, and future research:** | Discussion |
| **OTHER INFORMATION** | | |  |
| Registration and protocol | 24a | **Provide registration information for the review, including the register name and registration number, or state that the review was not registered.** | Just below the Abstract |
|  | 24b | **Indicate where the review protocol can be accessed, or state that a protocol was not prepared:** | Abstract and Methods |
|  | 24c | **Describe and explain any amendments to information provided at registration or in the protocol:** | NA |
| Support | 25 | **Describe sources of financial or non-financial support for the review, and the role of the funders or sponsors in the review** | Funding |
| Competing interests | 26 | **Declare any competing interests of review authors:** | Conflicts of interests |
| Availability of data, code and other materials | 27 | **Report which of the following are publicly available and where they can be found: template data collection forms; data extracted from included studies; data used for all analyses; analytic code; any other materials used in the review** | Data Availability Statement |
