# Supplementary material for: Age at menarche in South Asia: an interplay of sociodemographic, nutritional, lifestyle, anthropometric, biological, and environmental factors—a systematic review
Source: Front Public Health. 2026 Jul 15;14:1836422. doi: 10.3389/fpubh.2026.1836422 (PMC13415688; doi:10.3389/fpubh.2026.1836422)
Supplement: Supplementary file 2 [file Table_2.docx]

**SUPPLEMENTARY MATERIAL**

**Table S2**. Electronic Supplementary Material Appendix S2. Full search strategy

| **Database** | **Search terms** | **Results** |
| --- | --- | --- |
| PubMed | ("menarche"[MeSH] OR "age at menarche"[tiab] OR "menarcheal age"[tiab] OR "puberty"[MeSH] OR "sexual maturation"[MeSH]) AND (("body mass index"[MeSH] OR BMI[tiab] OR "overweight"[MeSH] OR obesity[MeSH] OR "nutritional status"[MeSH] OR "diet"[MeSH] OR "dietary patterns"[tiab] OR "physical activity"[MeSH] OR "sedentary behavior"[MeSH] OR "sleep"[MeSH] OR "screen time"[tiab]) OR ("socioeconomic factors"[MeSH] OR "urban population"[MeSH] OR "rural population"[MeSH] OR "educational status"[MeSH] OR "occupations"[MeSH] OR "family characteristics"[MeSH])) AND ("Afghanistan"[MeSH] OR "Bangladesh"[MeSH] OR "Bhutan"[MeSH] OR "India"[MeSH] OR "Nepal"[MeSH] OR "Pakistan"[MeSH] OR "Sri Lanka"[MeSH] OR "South Asia"[tiab]) | 230 |
| Web of sciences | TS= ((menarche OR "age at menarche" OR puberty ") AND (BMI OR "body mass index" OR overweight OR obesity OR diet OR "physical activity" OR sedentary OR sleep OR "screen time" OR socioeconomic OR urban) AND (India OR Pakistan OR Bangladesh OR Nepal OR Bhutan OR "Sri Lanka" OR Afghanistan)) | 204 |
| Science Direct | (menarche OR "age at menarche") AND (BMI OR diet OR "physical activity" OR socioeconomic) AND (India OR Pakistan OR Bangladesh OR Nepal OR Bhutan OR "Sri Lanka") | 161 |
| Google Scholar | "age at menarche" OR menarche OR puberty female children adolescent girls BMI Nutritional status diet physical activity socioeconomic India Pakistan Bangladesh Nepal Bhutan "Sri Lanka" "South Asia" | 134 |

*Record counts for all databases are as per the original search on date 15 July 2025. Due to ongoing database updates, the number of results may change over time. The original count is reported in the PRISMA flow diagram.*
