# Supplementary material for: Age at menarche in South Asia: an interplay of sociodemographic, nutritional, lifestyle, anthropometric, biological, and environmental factors—a systematic review
Source: Front Public Health. 2026 Jul 15;14:1836422. doi: 10.3389/fpubh.2026.1836422 (PMC13415688; doi:10.3389/fpubh.2026.1836422)
Supplement: Supplementary file 3 [file Table_3.docx]

**SUPPLEMENTARY MATERIAL**

**Table S3**. Quality assessment of cross-sectional studies by the Joanna Briggs Institute (JBI) checklists

| **Sr No** | **Study** | **Q1. Were the criteria for inclusion in the sample clearly defined?** | **Q2. Were the study subjects and the setting described in detail?** | **Q3. Was the exposure measured in a valid and reliable way?** | **Q4. Were objective, standard criteria used for measurement of the condition?** | **Q5. Were confounding factors identified?** | **Q6. Were strategies to deal with confounding factors stated?** | **Q7. Were the outcomes measured in a valid and reliable way?** | **Q8. Was appropriate statistical analysis used?** | **§Score** | **Rating** |
| --- | --- | --- | --- | --- | --- | --- | --- | --- | --- | --- | --- |
| 1 | Malitha 2020 | Yes | Yes | Unclear | No | Yes | Yes | Yes | Yes | 6 | Moderate |
| 2 | Islam 2017 | Yes | Yes | Yes | Yes | No | No | Unclear | Yes | 5 | Moderate |
| 3 | Dema 2019 | Yes | Yes | Yes | Yes | No | No | No | No | 4 | Low |
| 4 | Balamurugan 2024 | Yes | Yes | Yes | Yes | Unclear | No | Yes | Yes | 6 | Moderate |
| 5 | Sowjanya 2024 | Yes | Yes | Unclear | Unclear | Yes | Yes | No | Yes | 5 | Moderate |
| 6 | Agrawal 2020 | Yes | Yes | Yes | No | No | No | No | No | 3 | Low |
| 7 | Patil 2020 | Yes | Yes | Yes | Yes | Yes | Yes | Yes | Yes | 8 | High |
| 8 | Dharmarha 2020 | Yes | Yes | Unclear | Yes | No | No | Yes | Yes | 5 | Moderate |
| 9 | Singh 2020 | Yes | Yes | Yes | No | No | No | No | No | 3 | Low |
| 10 | Zeglen 2020 | Yes | Yes | Yes | Yes | Yes | Yes | No | Yes | 7 | High |
| 11 | Pandey 2017 | Yes | Yes | Yes | No | No | No | No | No | 3 | Low |
| 12 | Tarannum 2017 | Yes | Yes | Yes | Yes | No | No | No | No | 4 | Low |
| 13 | Goyal 2016 | Yes | Yes | Yes | Yes | No | No | No | No | 4 | Low |
| 14 | Chalise 2018 | Yes | Yes | Yes | No | No | No | No | No | 3 | Low |
| 15 | Tarar 2025 | Yes | Yes | Unclear | Yes | Yes | No | Unclear | No | 4 | Low |
| 16 | Karim 2021 | Yes | Yes | Unclear | Yes | Yes | Yes | Unclear | Yes | 6 | Moderate |
| 17 | Khalid 2015 | Yes | Yes | Yes | Unclear | N | No | Unclear | Yes | 4 | Low |

**§**Scoring: 0–3 = Low quality; 4–6 = Moderate quality; 7–10 = High quality
