# Supplementary material for: Age at menarche in South Asia: an interplay of sociodemographic, nutritional, lifestyle, anthropometric, biological, and environmental factors—a systematic review
Source: Front Public Health. 2026 Jul 15;14:1836422. doi: 10.3389/fpubh.2026.1836422 (PMC13415688; doi:10.3389/fpubh.2026.1836422)
Supplement: Supplementary file 4 [file Table_4.docx]

**SUPPLEMENTARY MATERIAL**

**Table S4.** Quality assessment of case-control study by Joanna Briggs Institute (JBI) checklists

| **Scoring scale for case control study** | **Bhattarai et al. (2018)** |
| --- | --- |
| Q1. Were the groups comparable other than presence of disease in cases or absence of disease in controls? | Yes |
| Q2. Were cases and controls matched appropriately? | No |
| Q3. Were the same criteria used for identification of cases and controls? | Yes |
| Q4. Was exposure measured in a standard, valid and reliable way? | Unclear |
| Q5. Was exposure measured in the same way for cases and controls? | Yes |
| Q6. Were confounding factors identified? | Yes |
| Q7. Were strategies to deal with confounding factors stated? | Yes |
| Q8. Were outcomes assessed in a standard, valid and reliable way for cases and controls? | Unclear |
| Q9. Was the exposure period of interest long enough to be meaningful? | Yes |
| Q10. Was appropriate statistical analysis used? | Yes |
| **§ Score** | **7** |
| **Rating** | **High** |

**§**Scoring: 0–3 = Low quality; 4–6 = Moderate quality; 7–10 = High quality
