# Supplementary material for: Age at menarche in South Asia: an interplay of sociodemographic, nutritional, lifestyle, anthropometric, biological, and environmental factors—a systematic review
Source: Front Public Health. 2026 Jul 15;14:1836422. doi: 10.3389/fpubh.2026.1836422 (PMC13415688; doi:10.3389/fpubh.2026.1836422)
Supplement: Supplementary file 5 [file Table_5.docx]

**SUPPLEMENTARY MATERIAL**

**Table S5.** Characteristics of the included research articles assessing the factors associated with age at menarche among females in studies selected from South Asian countries.

| **Sr. No.** | **First Author, year** | **JBI Rating** | **Sociodemographic factors** | | | | | | | **Nutrition & lifestyle factors** | | | | | **Anthropometric factors** | | | | | | | | | **Biological factors** | | | **Environmental factors** |
| --- | --- | --- | --- | --- | --- | --- | --- | --- | --- | --- | --- | --- | --- | --- | --- | --- | --- | --- | --- | --- | --- | --- | --- | --- | --- | --- | --- |
|  |  |  | **Residence** | **Economic status** | **⁋Type of school** | **^§^Parental characteristics** | **Caste/ religion** | **Family size** | **Birth order** | **Diet** | **Sleep** | **Physical activity** | **Psychosocial Stress** | **Screen time** | **Weight** | **Height** | **WC, HC & WHR** | **MUAC** | **HAZ** | **Stunting** | **UW** | **BMI** | **†Other** | **Birth weight** | **SAAM** | **MAAM** |  |
| **Bangladesh** | | | | | | | | | | | | | | | | | | | | | | | | | | | |
| 1 | Malitha 2020 (42) | M | ✔ | ✔ | × | ✔ | × | × | × | × | × | × | × | × | × | × | × | × | × | × | × | ✔ | × | × | × | × | × |
| 2 | Islam 2017 (48) | M | × | ✔ | ✔ | ✔ | ✔ | ✔ | × | × | × | × | × | × | × | × | × | × | × | × | × | × | × | × | × | × | × |
| **Bhutan** | | | | | | | | | | | | | | | | | | | | | | | | | | | |
| 3 | Dema 2019 (50) | L | × | ✔ | × | ✔ | × | ✔ | × | ✔ | × | × | × | × | × | × | × | × | × | × | × | ✔ | × | ✔ | × | × | × |
| **India** | | | | | | | | | | | | | | | | | | | | | | | | | | | |
| 4 | Balamurugan 2024 (37) | M | ✔ | × | × | × | × | × | × | ✔ | × | × | × | × | ✔ | ✔ | ✔ | × | × | × | × | ✔ | × | × | × | × | × |
| 5 | Sowjanya 2024 (38) | M | × | × | × | ✔ | × | × | × | ✔ | ✔ | ✔ | × | ✔ | × | × | × | × | × | × | × | ✔ | × | × | × | × | × |
| 6 | Agrawal 2022 (53) | L | × | × | × | × | × | × | × | × | × | × | × | × | × | × | × | × | × | × | × | ✔ | × | × | × | × | × |
| 7 | Patil 2020 (51) | H | × | × | × | × | × | × | × | × | × | × | × | × | ✔ | ✔ | ✔ | ✔ | × | ✔ | ✔ | ✔ | × | × | × | × | × |
| 8 | Dharmarha 2020 (46) | L | × | ✔ | × | × | × | × | × | × | × | × | × | × | × | × | × | × | × | × | × | × | × | ✔ | ✔ | × | × |
| 9 | Singh 2020 (45) | L | × | ✔ | × | × | × | × | × | × | × | ✔ | × | × | × | × | × | × | × | × | × | × | × | × | × | × | × |
| 10 | Zeglen 2020 (43) | H | × | ✔ | × | × | × | × | × | × | × | × | × | × | × | × | × | × | ✔ | × | × | ✔ | × | × | × | × | × |
| 11 | Pandey 2017 (49) | M | × | ✔ | × | × | × | × | ✔ | ✔ | × | × | × | × | × | × | × | × | × | × | × | ✔ | × | × | × | ✔ | × |
| 12 | Tarannum 2017 (47) | L | × | ✔ | × | ✔ | ✔ | ✔ | ✔ | × | × | × | × | × | × | × | × | × | × | × | × | × | × | × | × | × | × |
| 13 | Goyal 2016 (52) | L | × | × | × | × | × | × | × | × | × | × | × | × | ✔ | ✔ | × | × | × | × | × | × | ✔ | × | × | × | × |
| **Nepal** | | | | | | | | | | | | | | | | | | | | | | | | | | | |
| 14 | Bhattarai 2018 (54) | H | ✔ | × | ✔ | ✔ | × | ✔ | ✔ | ✔ | ✔ | ✔ | × | × | × | × | × | × | × | × | × | ✔ | × | ✔ | × | ✔ | ✔ |
| 15 | Chalise 2018 (39) | L | × | ✔ | × | × | ✔ | × | × | ✔ | × | × | × | × | ✔ | ✔ | × | × | × | × | × | ✔ | × | × | × | × | × |
| **Pakistan** | | | | | | | | | | | | | | | | | | | | | | | | | | | |
| 16 | Tarar 2025 (40) | L | × | ✔ | × | ✔ | × | ✔ | × | ✔ | × | ✔ | ✔ | × | × | × | × | × | × | × | × | ✔ | × | × | × | × | × |
| 17 | Karim 2021(44) | M | × | ✔ | ✔ | × | × | × | × | × | × | × | × | × | × | × | ✔ | × | × | ✔ | ✔ | ✔ | × | × | × | × | × |
| 18 | Khalid 2015(41) | L | ✔ | ✔ | × | × | × | × | × | × | × | ✔ | ✔ | × | × | × | × | × | × | × | × | ✔ | × | × | × | × | × |

✔: Parameter assessed; ×: parameter not assessed; ⁋:type of school board (central vs state board or public vs private)/ educational status of female adolescents; UW: Underweight; WC: Wasit circumference; HC: Hip circumference; WHR: Waist-to-Hip ratio; MUAC: mid-upper-arm-circumference; HAZ: Height-for-age; §Parental characteristics: parental education, occupation, presence or absence of biological parent; †Other anthropometric parameter: Larger skeletal frame measurements bi-acromial width, bi-iliac width, and arm span; SAAM: Sister’s age at menarche; MAAM: Mother’s age at menarche; JBI rating Joanna Briggs Institute (JBI) rating; H: High; M: Moderate; L: Low
