# Supplementary material for: Age at menarche in South Asia: an interplay of sociodemographic, nutritional, lifestyle, anthropometric, biological, and environmental factors—a systematic review
Source: Front Public Health. 2026 Jul 15;14:1836422. doi: 10.3389/fpubh.2026.1836422 (PMC13415688; doi:10.3389/fpubh.2026.1836422)
Supplement: Supplementary file 6 [file Table_6.docx]

**SUPPLEMENTARY MATERIAL**

**Table S6.** Association of Sociodemographic, Anthropometric, Nutritional, Lifestyle, Biological, and Environmental factors with age at menarche in females from South Asian countries

| **Sr. no.** | **First Author, year** | **Age (yrs).**  **City/Region; Sample size (N)** | **Mean AAM** | **Study design** | **DCT** | **Factors associated with age at menarche** | **Overall result** |
| --- | --- | --- | --- | --- | --- | --- | --- |
| Bangladesh | | | | | | | |
| 1 | Malitha 2020 (42) | 10–12; Rajshahi District; 386 | Not reported | Cross-sectional | SQ | Girl's BMI category:  Normal weight: AOR^a^ (95% CI): 0.279 (0.075–0.986);  p < 0.05  Overweight: AOR (95% CI): 0.594 (0.169–2.085) Obesity:1  Mother's occupation.  Housewife: AOR (95% CI): 0.753 (0.320–1.773)  Service: 1  Father's occupation:  Service: AOR (95% CI): 1.396 (0.402–4.843)  Business: AOR (95% CI): 1.281 (0.457–3.595)  Mother's education:  Uneducated: AOR (95% CI): 0.872 (0.116–6.569)  Primary: AOR (95% CI): 0.829 (0.232–2.964)  Secondary: AOR (95% CI): 0.889 (0.360–2.197)  Father's education:  Uneducated: AOR (95% CI): 4.698 (1.844–7.200)  Primary: AOR (95% CI): 3.125(1.632–5.451)  Secondary: AOR (95% CI): 2.030 (0.560–7.362)  Monthly family income:  <20,000 (Taka): AOR (95% CI): 1.499 (0.893–2.608)  > 20,000 (Taka)  Mode of birth:  Normal: AOR (95% CI): 1.142 (0.645–2.023)  Caesarean  Type of residence:  Rural: AOR (95% CI): 0.012 (0.003–0.047); p < 0.01  Urban R | The risk of early onset of menarche was higher in obese girls compared to those with normal BMI. Also, schoolgirls living in urban areas experienced menarche earlier than girls living in rural areas. |
| 2 | Islam 2017 (48) | 10–20; Jessore District; 24,898 | Mean ± SD 11.6 ± 3,6 years | Cross-sectional | SQ | Girl's Education:  5–7 grades: R  8–9 grades : AOR^⁋^ (95% CI): 2.74 (1.899–3.955)  10 grades : AOR (95% CI): 2.22 (1.482–3.323)  p < 0.0001  Marital status:  Married: R  Unmarried: AOR (95% CI): 2.42 (0.678–0.598)  p = 0.174  Type of family:  Nuclear: R; p = 0.638  Joint: AOR (95% CI): 0.77 (0.379–1.548); p = 0.457  Extended: AOR (95% CI): 1.08 (0.735–1.578) p = 0.704  Family size: 4 Persons : R.  4+ : AOR (95% CI): 1.13 (0.79–1.614); p = 0.506  Father’s education:  High school: R; p = 0.317  Graduate: AOR (95% CI): 1.17 (0.697–1.968); p = 0.551  Masters: AOR (95% CI): 1.50 (0.866–2.602); p = 0.148  Mother's education:  High school: R; p = 0.524  Graduate: AOR (95% CI): 0.76 (0.48–1.217); p = 0.258  Masters: AOR (95% CI): 0.85 (0.496–1.444); p = 0.541  Family income:  Up to 20,000 (BDT): R  > 20,000 (BDT): AOR (95% CI): 0.91 (0.636–1.314).  p = 0 626 | Higher education was associated with an earlier age at menarche |
| Bhutan | | | | | | | |
| 3 | Dema 2019 (50) | 9–18; Thimphu District; 286 | Mean ± SD 10.89 ± 1.618 years | Cross-sectional | SQ | BMI:  Overweight: R  Underweight: AOR^⁋^ (95% CI): 0.09 (0.02–0.57); p = 0.01  Normal weight: AOR (95% CI): 0.22 (0.04–1.31); p = 0.09  Age category:  12–18 years: R  9–12 years: AOR (95% CI): 0.12 (0.05–0.28); p < 0.0001  Mother’s occupation:  Employed: R  Farmer: AOR (95% CI): 1.43 (0.35–5.83); p = 0.61  Homemaker: AOR (95% CI): 2.09 (1.04–4.18); p = 0.04  Birth weight:  ELBW: R  Normal weight: AOR (95% CI): 0.68 (0.12–3.95); p = 0.67  Low BW: AOR (95% CI): 1.15 (0.17–9.24); p = 0.82  VLBW: AOR (95% CI): 0.00 (0.00–0.00); p = 0.99  Exclusively breastfed:  No response: R  Yes: AOR (95% CI): 1.15 (0.25–5.39); p = 0.87  No: AOR (95% CI): 1.48 (0.38–5.76); p = 0.57 | BMI, mother’s occupation and age were reported to be associated with age at menarche. However, overall result remains unclear as the author didn’t explicitly mention the list of variables adjusted for in the regression model |
| India | | | | | | | |
| 4 | Balamurugan 2024 (37) | 10–19; Coimbatore, Tamil Nadu; 790 | Mean ± SD 11.55 ± 1.06 years | Cross-sectional | SQ | Area of residence:  β Coeff (95 % CI): 0.264 (-0.955, -0.552); p = 0.001 Frequency of consumption of fruits:  β Coeff (95 % CI): -0.095 (-0.194, -0.023); p = 0.013 Frequency of consumption of green leafy vegetables:  β Coeff (95 % CI): -0.175 (-0.317, -0.096)  p = 0.0001 | An earlier age at menarche was observed in women residing in urban areas and among those with a higher frequency of fruit and vegetable consumption. |
| 5 | Sowjanya T 2024 (38) | 14–16; Karnataka; 700 daughters from  1071 mother-daughter pairs) | Mean ± SD 12.67 ± 1.19 years | Cross-sectional | SQ | Audio-visual media exposure: 1–3 hours/day: AOR^b^ (95% CI): 2.977 (1.644–5.394); p < 0.0001  ≥ 3 hours/day: AOR (95% CI): 1.459 (0.797–2.673); p *=* 0.221  < 1 hours/day: 1 Body Mass Index-for-age: <-2: AOR (95% CI): 0.486 (0.250–0.945); p = 0.033 ≥+1: AOR (95% CI): 2.606 (0.825–8.232); p = 0.103 -2 to+1: 1 Exercise: No: AOR (95% CI): 1.631 (0.978–2.718); p = 0.061 Yes: 1 Diet Non-vegetarian: AOR (95% CI): 2.710 (1.043–7.040); p = 0.041 Vegetarian: 1 Sleep time: Beyond 10 pm: AOR (95% CI): 2.448 (1.164–5.148); p = 0.018 9–10 pm: AOR (95% CI): 2.099 (1.043–4.224); p = 0.038 8–9 pm: 1 | High BMI, excessive exposure to audio-visual media, non-vegetarian diet and late sleeping habit were the risk factors of early menarche. |
| 6 | Agrawal 2022 (53) | 12–17; Durgapur, West Bengal; 600 | Mean ± SD 12.74 ± 1.07 years | Cross-sectional | SQ | BMI category kg/m^2:^ Mean ± SD AAM yr  < 18.5: Mean ± SD 12.92 ± 1.03  18.4–24.99: Mean ± SD 12.74 ± 1.07  > 25: Mean ± SD 12.60 ± 1.07  ANOVA-F: NR; p = 0.056 | No association was found between mean age at menarche and BMI. |
| 7 | Patil 2020 (51) | 10–17; Rural Maharashtra;  1071 | Mean ± SD 13.00 ± 1.1 years | Cross-sectional | SQ | Menarcheal age & Anthropometry  Stunting:  Age at menarche <12 years: 1 Age at menarche <13 years:  OR (95% CI): 1.67 (0.70–3.98); p = 0.24 Age at menarche <14 years:  OR(95% CI): 2.24 (0.97– 5.20); p = 0.059 Age at menarche <15 years:  OR(95% CI): 1.89 (0.80– 4.48); p = 0.15 Age at menarche ≥15 years:  OR(95% CI): 3.43 (1.29– 9.11); p = 0.013 Thin:  Age at menarche <12 years: 1 Age at menarche <13 years:  OR(95% CI): 3.65 (1.08–12.4); p < 0.0001 Age at menarche <14 years:  OR(95% CI): 7.25 (2.20–23.89); p < 0.0001  Age at menarche <15 years:  OR(95% CI): 10.69 (3.22–35.46); p = 0.001 Age at menarche ≥15 years:  OR(95% CI): 12.43 (3.42–45.1); p = 0.04 Overweight:  Age at menarche <12 years: 1 Age at menarche <13 years:  OR(95% CI):0.38 (0.13– 1.11); p = 0.08 Age at menarche <14 years:  OR(95% CI): 0.13 (0.04–0.43); p = 0.001  Age at menarche <15 years:  OR (95% CI): 0.29 (0.09–0.87); p = 0.027 Age at menarche ≥15 years:  OR (95% CI): 0.14 (0.02– 1.24); p = 0.078 | An increasing age at menarche was associated with higher odds of stunting and thinness while decreasing age at menarche was associated with higher odds of overweight. |
| 8 | Dharmarha 2018 (46) | 9–16; Mumbai Maharashtra; 258 | Mean ± SD 12.23 ± 1.09 years | Cross-sectional | SQ | Sister's age at menarche: n (%) 10–12: 34 (28.1)  12–14: 61 (50.4)  14–16: 26 (21.5)   p = 0.002 Socioeconomic status High (Class I and II): n (%) ≤ 12: 52 (23.2)  > 12: 172 (76.8) Low (Class III and IV): 1.81 ≤ 12: 02 (5.9)  > 12: 32 (94.1)  p = 0.021 | Sister's age at menarche and socioeconomic status were associated with early age at menarche |
| 9 | Singh 2020 (45) | 13–18; Kangra district of Himachal Pradesh; 276 | Mean age: 12.75 yrs | Cross-sectional | SQ | Physical activity and menarche Early menarche n (%) High physical activity: 92 (33.33)  Moderate physical activity: 17 (6.15) Low physical activity: 5 (1.81) Medium menarcheal age n (%) High physical activity: 26 (9.42) Moderate physical activity: 57 (20.65) Low physical activity: 10 (3.62) Delayed menarcheal age n (%) High physical activity: 7 (2.53) Moderate physical activity: 14 (5.09) Low physical activity: 49 (7.4 )  χ² = 183.939; p < 0.01  Socioeconomic status and menarche Early menarche n (%) High/Upper: 99 (35.9) Medium/Middle: 10 (3.62) Low/Lower: 5 (1.81) Median menarcheal age n (%) High/Upper: 8 (29.7) Medium/Middle: 6 (2.17) Low/Lower: 5 (1.81) Delayed menarcheal age n (%) High/Upper: 39 (14.13) Medium/Middle: 21 (7.6) Low/Lower: 9 (3.26)  χ² = 31.467; p < 0.01 | Adolescent girls who were in better socio-economic category and were physically more active attained menarche at an earlier age in comparison to others. |
| 10 | Zeglen 2020 (43) | 7–21; Kolkata city; 2195 | Mean ± SD  11.8 ± 1.2 | Cross-sectional study | SQ | Survival probability value (BMI)  0.68 (BMI: 25-35)  Survival probability value (HAZ)  0.70 (HAZ: 2)  Survival probability value (socio-economic variables)  0.67 | Early menarche probability increased with increasing BMI, height-for-age z-scores, household size, number of rooms, and toilet availability. |
| 11 | Pandey 2017 (49) | 10–19; Sikkim; 430 | Mean ± SD  13.64 ± 1.58 | Cross-sectional study | SQ | Socioeconomic class: AAM: n (%) Upper: 10–11 yrs: 61(65.5%); 12–14yrs: 112 (36.7%); ≥ 15: 0 Upper middle:10–11 yrs: 27 (29%); 12–14yrs: 90(29.5%); ≥ 15: 6 (18.8%) Lower middle: 10–11 yrs: 3 (3.2%); 12–14yrs: 70 (23%); ≥ 15: 16 (50%) Upper lower: 10–11 yrs: 2 (2.2%); 12–14yrs: 33(10.8%); ≥ 15: 10 (31.3%) p < 0.001 Mother’s age at menarche: AAM: n(%) 1-10: 10–11 yrs: 0; 12–14yrs: 2(0.7%); ≥15: 0 11-15:10–11 yrs: 93(100%); 12–14yrs: 272(89.2%); ≥15: 8(25%) 16-20: 10–11 yrs: 0; 12–14yrs: 31(10.2%); ≥15: 24(75%) Mother’s age at menarche: (r = 0.549); p < 0.001 BMI: AAM: n(%) Undernutrition: 10–11 yrs: 2(2.2%); 12–14 yrs: 10(3.3%); ≥15: 1(3.1%) Normal wt:10–11 yrs: 60(64.5%); 12–14yrs: 240(78.7%); ≥15: 28(87.5%) Overweight: 10–11 yrs: 18(19.4%); 12–14yrs: 34(11.1%); ≥15: 3(9.4%) Obesity: 10–11 yrs: 13(14%); 12–14yrs: 21(6.9%); ≥15: 0 Body mass index: (r = -0.155); p = 0.029  Diet: AAM: n (%)  Veg: 10–11 yrs: 10(10.8%); 12–14yrs: 34(11.1%); ≥15: 5(15.6%)  Non-veg: 10–11 yrs: 83(89.2%); 12–14yrs: 271(88.9%); ≥15: 27(84.4%); p = 0.732 Birth order: AAM: n (%) 1^st^: 10–11 yrs: 52(55.9%); 12–14yrs: 142(46.6%); ≥15: 13(40.6%) 2^nd^: 10–11 yrs: 29(31.2%); 12–14yrs: 89(29.2%); ≥15: 9(28.1%)  3^rd^: 10–11 yrs: 9(9.7%); 12–14yrs: 40(13.1%); ≥15: 3(9.4%)  ≥4^th^: 10–11 yrs: 3(3.2%); 12–14yrs: 33(10.8%); ≥15: 7(21.9%); p = 0.069 | Higher BMI was associated with an earlier age at menarche, which was also observed among girls from lower socioeconomic classes. Additionally, daughters tended to experience menarche at ages similar to those of their mothers. |
| 12 | Tarannum 2017 (47) | 12–14; Aligarh, Uttar Pradesh; 422 | Mean ± SD 12.52 ± 1.41 years | Cross-sectional | SQ | Menarcheal age: Religion Islam: Mean ± SD 12.52 ± 1.37 Hindu: Mean ± SD 12.52 ± 1.59 t:0.005; p = 0.996 Menarcheal age: Family size < 5: Mean ± SD 12.40 ± 1.24 5–10: Mean ± SD 12.55 ± 1.48 > 10: Mean ± SD 12.79 ± 1.70 F:1.906; p = 0.150  Menarcheal age: Birth order 1–2: Mean ± SD 12.21 ± 1.35 3–4: Mean ± SD 12.77 ± 1.26 > 4: Mean ± SD 12.79 ± 1.79 F:8.485; p < 0.0001 Menarcheal age: Education status of father Illiterate: Mean ± SD 13.00 ± 1.23 Primary: Mean ± SD 12.25 ± 1.86 Middle: Mean ± SD 12.06 ± 1.36 High school: Mean ± SD 12.64 ± 1.63 Intermediate: Mean ± SD 12.45 ± 1.21 Graduate and above: Mean ± SD 12.54 ± 1.39 F:1.249; p = 0.285  Menarcheal age: Education status of mother Illiterate: Mean ± SD 12.78 ± 1.45 Primary: Mean ± SD 12.14 ± 1.55 Middle: Mean ± SD 12.31 ± 1.60 High school: Mean ± SD 12.48 ± 1.42 Intermediate: Mean ± SD 12.57 ± 1.26 Graduate and above: Mean ± SD 12.53 ± 1.40 F:0.837; p = 0.524  Menarcheal age: Socioeconomic status Class 1: Mean ± SD 12.30 ± 1.37 Class 2: Mean ± SD 12.45 ± 1.33 Class 3: Mean ± SD 12.67 ± 1.52 Class 4: Mean ± SD 12.86 ± 1.42 Class 5: Mean ± SD 13.55 ± 1.58 F:4.346; p = 0.002 | Girls of lower birth order experienced menarche at an earlier age compared to those born third or fourth in order. |
| 13 | Goyal 2016 (52) | 11–14; Punjab; 200 | 12.3 years | Cross-sectional | SQ | Menarcheal age: Body weight (kg)  11yrs: Mean ± SD 43.99 ± 4.16 12yrs: Mean ± SD 41.22 ± 4.08 13yrs: Mean ± SD 40.59 ± 3.63 14yrs: Mean ± SD 40.94 ± 2.53 Correlation coefficient:-0.073; p = 0.122 Menarcheal age: Height (cms)  11yrs: Mean ± SD 146.66 ± 8.73 12yrs: Mean ± SD 149.46 ± 3.94 13yrs: Mean ± SD 149.94 ± 4.24 14yrs: Mean ± SD 153.19 ± 4.67 Correlation coefficient:0.170; p = 0.024 Menarcheal age: Biacromial width (cms)  11yrs: Mean ± SD 31.55 ± 1.47 12yrs: Mean ± SD 31.73 ± 1.69 13yrs: Mean ± SD 32.43 ± 1.78 14yrs: Mean ± SD 33.53 ± 1.18 Correlation coefficient:0.310; p = 0.003 Menarcheal age: Bi-iliac width (cms) 11yrs: Mean ± SD 24.35 ± 1.67 12yrs: Mean ± SD 24.31 ± 1.96 13yrs: Mean ± SD 25.90 ± 1.88 14yrs: Mean ± SD 24.73 ± 1.83 Correlation coefficient:0.252; p = 0.048 Menarcheal age: Arm span (cms) 11yrs: Mean ± SD 143.99 ± 9.25 12yrs: Mean ± SD 141.22 ± 4.27 13yrs: Mean ± SD 140.59 ± 4.07 14yrs: Mean ± SD 140.94 ± 4.68 Correlation coefficient:0.198; p = 0.009 | Height, bi-acromial width, bi-iliac width and arm span were associated with the age of menarche in adolescent girls. |
| Nepal | | | | | | | |
| 14 | Bhattarai 2018 (54) | 10–12; Pokhara; 260(130 cases and 130 controls | Menarcheal age in cases: Mean ± SD 10.82 ± 0.5 years Menarcheal age in control: Mean ± SD 14.26 ± 0.7 years | Case-control | SQ | Physical inactivity AOR^c^ (95% CI): 5.694 (1.932–16.787); p < 0.001 Low birth weight AOR (95% CI): 9.444 (3.022–29.517); p = 0.002 Inadequate sleep AOR (95% CI): 8.077 (2.628–24.830); p = 0.001 Absence of biological father AOR (95% CI): 10.001 (1.195–83.660); p = 0.034 Exposure to sexual material AOR (95% CI): 34.782 (6.366–190.026); p = 0.008 Inadequate breastfeeding AOR (95% CI): 5.204 (1.544–17.543); p = 0.001 Mother's age at menarche AOR (95% CI): 10.782 (3.381–34.389); p < 0.001 Use of hormonal contraceptives by mother AOR (95% CI): 5.805 (1.722–19.568); p = 0.005 Exposure to chemicals during pregnancy AOR (95% CI): 3.917 (1.159–13.234); p = 0.028 | Physical inactivity, low birth weight, inadequate sleep, absence of biological father, exposure to sexual material, inadequate breastfeeding, mother's age at menarche, use of hormonal contraceptive by mother, and exposure to chemicals during pregnancy were associated with early menarche |
| 15 | Chalise 2018 (39) | 9–18; Pokhara; 500 | Mean ± SD  12.56 ± 1.12 | Cross-sectional | SQ | Menarcheal age: Dietary habits Veg: Mean ± SD 12.41 ± 1.06 Non-veg: Mean ± SD 12.59 ± 1.14 t:-1.106 ; p = 0.37 Menarcheal age: Income < 30 thousands: Mean ± SD 12.59 ± 1.13 30 thousands and above: Mean ± SD 12.50 ± 1.11 t:0.44; p = 0.44 Menarcheal age: Race Brahmin: Mean ± SD 12.67 ± 1.16 Newar: Mean ± SD 12.31 ± 1.47 Mongoloids: Mean ± SD 12.52 ± 1.07  Others: Mean ± SD 12.61 ± 1.02 F:1.27; p = 0.28 | No association was found between dietary habits, family income and race with the mean age at menarche. |
| Pakistan | |  |  |  |  |  |  |
| 16 | Tarar 2025 (40) | 9–15; Urban and rural areas of Karachi; 386 | Mean ± SD  11.93 ± 1.08 | Cross-sectional | SQ | Menarcheal age: BMI category (Frequency) 9yrs: UW (0); Healthy weight (14); Overweight (1); Obese:(0) 10yrs: UW (14); Healthy weight (8); Overweight (3); Obese:(2) 11yrs: UW (30); Healthy weight (26); Overweight (6); Obese:(0) 12yrs: UW (71); Healthy weight (47); Overweight (4); Obese:(0) 13yrs: UW (39); Healthy weight (29); Overweight (5); Obese:(0) 14yrs: UW (4); Healthy weight (6); Overweight (1); Obese:(1) 15yrs: UW (1); Healthy weight (2); Overweight (0); Obese:(0) p = 0.024 Menarcheal age: Father's occupation (Frequency) 9yrs: Businessman (0); Doctor (1); Labourer (0); Other (0);  Private job (3); Public job (0); Scientist (0) 10yrs: Businessman (5); Doctor (1); Labourer (5); Other (2);  Private job (10); Public job (4); Scientist (1) 11yrs: Businessman (11); Doctor (0); Labourer (2); Other (6);  Private job (32); Public job (10); Scientist (1) 12yrs: Businessman (34); Doctor (3); Labourer (10); Other (9);  Private job (40); Public job (18); Scientist (8) 13yrs: Businessman (13); Doctor (3); Labourer (9); Other (6);  Private job (24); Public job (13); Scientist (5) 14yrs: Businessman (2); Doctor (0); Labourer (5); Other (0);  Private job (4); Public job (1); Scientist (0) 15yrs: Businessman (0); Doctor (0); Labourer (3); Other (0);  Private job (0); Public job (0); Scientist (0) p = 0.003 | Early menarche was observed in girls with higher BMI and among those whose fathers worked in private sector jobs. |
| 17 | Karim 2021 (44) | 8–16; Punjab province of Pakistan; 10,050 | Mean ± SD 12.4 years | Cross-sectional | SQ | Low socioeconomic status 12 yrs: Coeff (SE): 1.555 (0.816); p < 0.05 16 yrs: Coeff (SE): -0.216 (0.787); p < 0.001  Stunting, 9–16 yrs: Coeff (SE) : -0.114 (0.471); p > 0.05  Underweight,  9–16 yrs: Coeff (SE) : 2.807 (0.536); p < 0.001  13 yrs: Coeff (SE): 2.708 (1.094); p < 0.05 14 yrs: Coeff (SE): 5.142 (1.077); p < 0.001  15 yrs: Coeff (SE): 3.768 (1.093); p < 0.001  Overweight, 9–16 yrs: Coeff (SE) : -2.555 (0.431); p < 0.001  13 yrs: Coeff (SE): -2.263 (0.764); p < 0.01 14 yrs: Coeff (SE): -3.787 (0.874); p < 0.001 | Girls belonging to low socioeconomic status (SES) experienced delayed onset of menarche compared to those from middle or high SES, while early onset of menarche was observed in overweight or obese girls compared to their lean counterparts. |
| 18 | Khalid 2015 (41) | 13–17; Lahore; 199 | Mean ± SD  12.66 ± 1.12 | Cross-sectional | SQ | Menarcheal age: Place of residence Peri-urban: Mean AAM 12.92  Urban: Mean AAM 12.37  Mean difference 0.55; p < 0.0001  Menarcheal age: Socioeconomic status Low: Mean AAM 13.06  Not low: Mean AAM 12.54  Mean difference 0.52; p = 0.004  Menarcheal age: BMI Undernourished: Mean AAM 12.79  Undernourished: Mean AAM 12.52  Mean difference 0.27; p = 0.096  Menarcheal age: Stress  Mild: Mean AAM 12.69  Moderate to heavy: Mean AAM 12.67  Mean difference 0.26; p = 0.937  Menarcheal age: Physical activity  Mild: Mean AAM 13.50  Moderate to heavy: Mean AAM 12.66  Mean difference 0.84; p = 0.294 | Age at menarche were associated with place of residence and socioeconomic status but no associations were found with stress, physical activity and BMI. |

DCT: Data Collection Tool; SQ: Structure Questionnaire; SE: Standard Error; BMI: Body mass index; Coeff (SE): Co-efficient (Standard Error); AOR: Adjusted Odds Ratio; COR: Crude Odds Ratio’s; SE: Standard Error; OR: Odds Ratio; UW: Underweight; Yrs: Years; AOR^a^: Adjusted for Girl's BMI Category, monthly family income, maternal age and stature, parental Occupation and education, mode of birth, type of residence; AOR^b^: Audio-visual media expo-sure, body mass Index-for-age, exercise, diet, sleep time; AOR^c^: Sleeping hours, physical activity, birthweight, breastfeeding duration, exposure to sexual materials, maternal age at menarche, use of hormonal contraceptive, chemical exposure during pregnancy, absence of biological father; AAM: Age at menarche; R: Reference category; BDT: Bangladesh Taka; AOR^⁋^: The covariate adjusted for in the multivariate model were not reported by the study
